# Chronotype, circadian rhythm, and psychiatric disorders: Recent evidence and potential mechanisms

**DOI:** 10.3389/fnins.2022.811771

**Published:** 2022-08-10

**Authors:** Haowen Zou, Hongliang Zhou, Rui Yan, Zhijian Yao, Qing Lu

**Affiliations:** ^1^Nanjing Brain Hospital, Medical School, Nanjing University, Nanjing, China; ^2^Department of Psychiatry, The Affiliated Brain Hospital of Nanjing Medical University, Nanjing, China; ^3^School of Biological Sciences and Medical Engineering, Southeast University, Nanjing, China; ^4^Child Development and Learning Science, Key Laboratory of Ministry of Education, Nanjing, China

**Keywords:** chronotype, circadian rhythm, psychiatric disorders, depression, sleep disorder

## Abstract

The circadian rhythm is crucial for physiological and behavioral functions. Chronotype, which represents individual preferences for activity and performance, is associated with human health issues, particularly psychiatric disorders. This narrative review, which focuses on the relationship between chronotype and mental disorders, provides an insight into the potential mechanism. Recent evidence indicates that (1) the evening chronotype is a risk factor for depressive disorders and substance use disorders, whereas the morning chronotype is a protective factor. (2) Evening chronotype individuals with bipolar disorder tend to have more severe symptoms and comorbidities. (3) The evening chronotype is only related to anxiety symptoms. (4) The relationship between chronotype and schizophrenia remains unclear, despite increasing evidence on their link. (5) The evening chronotype is significantly associated with eating disorders, with the majority of studies have focused on binge eating disorders. Furthermore, the underlying mechanisms or influence factors are described in detail, including clock genes, brain characteristics, neuroendocrinology, the light/dark cycle, social factors, psychological factors, and sleep disorders. These findings provide the latest evidence on chronotypes and psychiatric disorders and serve as a valuable reference for researchers.

## Introduction

Circadian rhythms are driven by the internal biological clock, producing 24-h rhythm autonomously while being synchronized daily by environmental signals (zeitgebers). Chronotype reflects the individual variability in the phase of entrainment. In the modern world, social activities may not be consistent with personal preference for sleep–wake time, disrupting our normal circadian rhythm, known as circadian misalignment or social jet lag (SJL). Researchers have found that circadian misalignment may be associated with medical illnesses, like neurodevelopmental disorders, psychiatric disorders, neurodegenerative disorders, cardiac diseases, and cancers (Germain and Kupfer, 2008; Logan and McClung, 2019).

This narrative review aims to summarize recent studies on chronotype and examine its relationship with mental disorders. The structure is as follows. We first described the definition and relation of circadian rhythms, sleep regulation, chronotype, and SJL to clarify the potential mechanism and the relationship to human health. Then, we reviewed the differences between chronotypes and the relationship with psychiatric disorders. In the third section, we discussed the relationship between chronotype and psychiatric disorders in detail, including depressive disorders, bipolar disorders (BD), anxiety disorders, schizophrenia, substance use disorders, and eating disorders. Finally, we concluded with a summary of the current state of research, identifying gaps and providing suggestions regarding future research.

### Circadian rhythm

The word “circadian,” meaning a cycle across one day, can be deconstructed as “circa” (meaning “about”) and “dies or diem” (meaning “a day”) (Bauducco et al., 2020). The physiological parameters, behavioral performance, and cognitive functions of humans vary during the 24-h day (Croce et al., 2018). Circadian rhythms, which are defined as 24-h biological rhythms, are driven by endogenous oscillators (such as suprachiasmatic nuclei and pineal and peripheral oscillators), and exogenous factors (such as the light/dark cycle, social life, and the sleep-wake cycle) which can synchronize the biological clock. To be precise, those periodic exogenous factors, called “zeitgeber” (“time giver” in German), are the environmental time cues that are likely to synchronize/entrain circadian rhythms every day (Golombek and Rosenstein, 2010). The relationship through which the circadian oscillator synchronizes with zeitgebers in phase or period is called the entrainment. It is a necessary process since the period of the endogenous oscillators is close to but not exactly 24 h, which may result in a drift or desynchronization with the solar rhythm without being regularly reset by zeitgebers. The phase of entrainment depends on the intrinsic period of the circadian oscillator and the strength of the zeitgeber. The strength comprises the range of intensities and sensitivity of the circadian oscillator to the zeitgeber (Aschoff and Pohl, 1978).

The master endogenous oscillator is situated in the hypothalamic suprachiasmatic nuclei (SCN), which receives light signals through melanopsin-containing photoreceptors in the retinal ganglion cells (Logan and McClung, 2019). The SCN is an essential central pacemaker that produces and coordinates circadian rhythms in organisms and is known as the master clock or circadian oscillator in mammals. After the retinal ganglion cells are integrated, the external light signals project through the retinohypothalamic tract to the SCN via the optic chiasm (Etain et al., 2011; Jagannath et al., 2017). At the cellular-molecular level, the clock or oscillator is driven by a negative and transcriptional-translational feedback loop of clock genes (described in section “Clock genes and their polymorphisms play a role in phenotypes and are linked with psychiatric disorders”). The SCN is responsible for receiving and integrating information from the zeitgeber, regulating the functions of other brain areas and organs, and coordinating the body to maintain rhythms consistent with the external environment (Jagannath et al., 2017).

### Sleep regulation

In humans, two intrinsic oscillators, namely the circadian oscillator (Process C) and the homeostatic oscillator (Process S), regulate two aspects of sleep (sleep duration and sleep time, respectively) (Gruber and Cassoff, 2014). In Process C, the circadian oscillator produces the waking drive, which increases during the daytime and reaches maximum intensity at the end of the day, and the sleep drive, which increases during the night and reaches a maximum at the end of the night (Taillard et al., 2021). This oscillator regulates sleep timing but not sleep duration. In Process S, the homeostatic oscillator generates homeostatic sleep pressure that increases during wakefulness and reduces during sleep. Regarding the mechanism of sleep pressure, the hypothesis is that the accumulation of neurotransmitters (such as adenosine) leads to neuronal activation during wakefulness (Dijk and von Schantz, 2005). This oscillator mainly regulates the depth and length of sleep. During a biological day, sleep pressure is counteracted by the drive of wakefulness to remain awake. Drowsiness occurs when the sleep pressure increases while the waking drive rapidly dissipates. After sleep onset, the sleep pressure begins to dissipate, but its effect is replaced by the increased circadian drive of sleep, which extends the sleep period. The dissipation of the sleep drive leads to the desire to wake up (Dijk and von Schantz, 2005).

In addition, orexin and orexin receptors play a crucial role in regulating sleep and wakefulness. The neurons in the lateral hypothalamic area (LHA) produce orexin A and B (regulators of feeding behavior), which can activate monoaminergic and cholinergic neurons in the hypothalamus and brain stem. These monoaminergic neurons drive arousal and wakefulness by producing norepinephrine, serotonin, dopamine, or histamine (Scammell et al., 2017). During the daytime, the SCN indirectly activates LHA, leading to these monoaminergic cells sending excitatory projections to the thalamus cortex and inhibiting the ventrolateral preoptic area (VLPO). During sleep, VLPO may be activated by sleep substances (e.g., adenosine) and inhibits monoaminergic cells and neurons in LHA to maintain sleep (Sakurai, 2007).

### Chronotype

Chronotype, referred to as diurnal preference, is a part of circadian rhythmicity (Adan et al., 2012) and is typically defined as individual variation in the preferred timing of the sleep–wake cycle (Zavada et al., 2005). More precisely, chronotype reflects individual variability in the phase of entrainment and is associated with variations of physiological nature, such as the rhythm of body temperature and hormone secretion (Taylor and Hasler, 2018). Chronotype is evaluated using self-reported questionnaires, usually the Morningness-Eveningness Questionnaire (MEQ) (Horne and Ostberg, 1976), its reduced five-item version (rMEQ) (Adan and Almirall, 1991), and the Munich Chronotype Questionnaire (MCTQ) (Roenneberg et al., 2003). The Composite Scale of Morningness (CSM) (Smith et al., 1989), the Diurnal Type Scale (DTS), the Circadian Type Questionnaire (CTQ), and the Preference Scale (PS) can also be considered instruments for evaluating chronotype (Adan et al., 2012). Other relatively objective tools can evaluate chronotypes, including core body temperature, dim light melatonin onset (DLMO), a sleep diary (Carney et al., 2012), wrist accelerometry, and actigraphs (Kaufmann et al., 2018; Lyall et al., 2018). We have reviewed the three most commonly used questionnaires in the Supplementary material.

Recent studies commonly divide chronotypes into three (or five) types: morning chronotype (moderate morning chronotype and definite morning chronotype), evening chronotype (moderate evening chronotype and definite evening chronotype), and intermediate chronotype (Horne and Ostberg, 1976). Morning chronotype individuals, also known as early chronotypes or larks, prefer to be active in the morning and sleep and wake early. Evening chronotypes, also called late chronotypes or owls, prefer to be active in the evening and sleep and wake up late. The intermediate chronotype (neutral or neither type) has no preference for morning or evening. Morning chronotype individuals achieve peak physical and mental performance in the early part of the day after waking up. Conversely, evening chronotype individuals have the best mental and physical performance before sleeping (Song et al., 2018). Most research indicated that the circadian phase of the morning chronotype is advanced by two or three hours than the evening chronotype by measuring the core temperature or melatonin (Baehr et al., 2000; Lack et al., 2009; Taillard et al., 2011). In addition, evening chronotype is also related to larger daily sleep debt, a great need for sleep, and morning sleepiness (Taillard et al., 1999, 2004). Taillard et al. investigated the effect of chronotype on the sleep-wake cycle in a large and demographic matching sample of all ages (617 subjects between 17 and 80 years). This study has shown that evening chronotypes are related to greater subjective sleepiness but have no difference in sleep duration, which presents significant daily sleep debt during the week in the eveningness. In addition, evening chronotypes show more variable sleep–wake habits and greater caffeine consumption (Taillard et al., 1999).

There are two main hypotheses to explain the differences between chronotypes. The first is the difference in the intrinsic circadian period between chronotypes. Single mutations in clock genes may contribute to significant variation in the intrinsic circadian period (Gentry et al., 2021). The intrinsic period or the period of the endogenous oscillators is not exactly 24 h and will be entrained by zeitgeber every day. Hence, a short intrinsic period is related to a rapid biological clock resulting in phase advance or morning chronotype with time elapsing. Conversely, a long intrinsic period eventually leads to the evening chronotype (Taillard et al., 2021). The lengths of intrinsic period are 24.3 h in the evening chronotype to 24.1 h in the morning chronotype (Lack et al., 2009). These results demonstrate a specific relationship between the properties of the circadian oscillator (the intrinsic circadian period) and chronotype (variability in the circadian phase) (Duffy et al., 2001). Indeed, this hypothesis included two aspects that could affect the behavior difference between chronotypes: genetics and zeitgebers.

Furthermore, the kinetic differences in the accumulation of sleep pressure in process S are another possible mechanism. The evening chronotype has a slower sleep pressure accumulation when awake and a slower decrease during sleep, which leads to relative sleepiness rhythm delays (Taillard et al., 2003; Lack et al., 2009). The homeostatic oscillator is interactive with the circadian oscillator: the intensity of sleep pressure is negatively related to the amplitude of the circadian rhythm (Taillard et al., 2021). These kinetic differences between chronotypes lead to differences in sleep parameters, especially the strength of subjective sleepiness. Most researchers indicated higher subjective sleepiness in the evening chronotype than the morning (Taillard et al., 1999, 2011). Among young people, the evening chronotype tends to wake up later because they have higher subjective sleepiness (5–9 h) than the morning chronotype (Lack et al., 2009). Interestingly, there is no difference in daytime objective alertness and performance between chronotypes though the evening chronotypes have greater subjective sleepiness (Taillard et al., 2011).

The internal mechanism of chronotype includes the interaction between the circadian process (affected by genetics and zeitgebers) and the sleep homeostatic process (driven by the accumulation and dissipation of neurotransmitters). According to Roenneberg et al. (2019), the chronotype reflects a stable state of individuals but not a trait because it varies under different strengths of zeitgeber. Nonetheless, chronotypes have high stability among individuals in real-world situations (McHill et al., 2021). This stability is so strong that the chronotype will relapse once the intervention of light exposure stops (Zerbini et al., 2020). It is important to understand that the essence of chronotype is state or trait-like, although not a trait exactly.

### Chronotype, social jet lag (circadian misalignment), and health

The evening chronotype has been associated with physical and psychological health problems, when the internal chronotype and external environment desynchronize. Some mental disorders have been associated with evening chronotype, including MDD, BD, SAD, anxiety disorders, psychotic disorders, and addictive disorders (Jagannath et al., 2017; Taylor and Hasler, 2018). Evening chronotype was associated with depressive and anxious symptoms, habitual use of alcohol and tobacco, and an increased risk of suicidality (Gau et al., 2007; Gaspar-Barba et al., 2009). On the other hand, some mental disorders may influence the association between chronotypes and other illnesses or specific behaviors. Depression and anxiety fully mediated the relationship between evening chronotype and cyberbullying perpetration (Tosuntas et al., 2018). In terms of marital satisfaction, chronotype also has a predictive role, with the morning chronotype having higher marital satisfaction (Shareh and Eshaghi Sani, 2019). Chronotype has been associated with nervous system diseases such as dementia, fibromyalgia (Thapa et al., 2020; Turkoglu and Selvi, 2020), and epilepsy (Manni et al., 2016), or physiological factors such as adiposity (Cespedes Feliciano et al., 2019).

Since exogenous factors (e.g., school schedule, shift work, and jet lag) exist, there is a misalignment between activity and sleep time between life rhythmicity and the biological clock, usually known as circadian rhythm disruption or SJL. SJL is commonly calculated as the absolute value of the difference in the midpoint of sleep time between weekdays and weekends using MCTQ (Henderson et al., 2019). It is a measure to describe and quantify circadian rhythm disruption, which is described as an abnormal phase angle difference between two or more rhythms. Specifically, SJL quantifies the circadian misalignment of the biological and social clocks only (Wittmann et al., 2006). Disrupted circadian rhythmicity or SJL may be crucial in the development, outcome, and treatment of human health (Foster et al., 2013; Jagannath et al., 2017; Gold and Kinrys, 2019). A study of more than 90,000 participants indicated that circadian dysfunction is related to a range of mental disorders, such as major depressive disorder (MDD), BD, and seasonal affective disorder (SAD), as well as to poorer mental experiences, such as more loneliness, lower happiness, and reduced satisfaction (Lyall et al., 2018). Research using unpredictable chronic mild stress in a mice model with depression-like behaviors identified decreased amplitude of activity and body temperature rhythm (Logan et al., 2015). Association between SJL and a higher risk of depression was found in both shift and non-shift workers (Lee et al., 2017; Islam et al., 2020). In addition, SJL and evening chronotype are related to metabolic disorders in adolescent girls (Cespedes Feliciano et al., 2019).

The evening chronotypes show larger SJL because it accumulates greater sleep debt during the weekdays, which results in later and longer sleep during weekends as compensation (Roenneberg et al., 2019). Since the development of sociality (use of artificial light and alarm clocks), solar zeitgebers have decreased influence on human circadian rhythms, whereas social zeitgebers have become more significant (Borisenkov et al., 2019). Compared to other ages, teenagers and young adults show the largest SJL (Foster et al., 2013) since the school schedule is more adaptive to morning chronotype. In contrast, the proportion of evening chronotype in teenagers and young adults is large. The conclusion that evening chronotype is more likely to have circadian misalignment gives us a clue as to why evening chronotype is associated with psychological health in humans.

## The differences between chronotypes and the association with psychiatric disorders

### Clock genes and their polymorphisms play a role in phenotypes and are linked with psychiatric disorders

A series of studies showed that clock genes contribute to the association between chronotype and mental health (Taylor and Hasler, 2018). In humans, *Brain and Muscle ARNT-like 1* (BMAL1) and *Circadian Locomotor Output Cycles Kaput Gene* (CLOCK) proteins form heterodimers, Basic Helix-Loop-Helix -Per-Arnt-sim (BHLH-PAS), through their PAS domain and bind to the E-box vis the BHLH domain, enhancing unit of the *Period gene* (PER1–3) and *Cryptochrome* (CRY1, 2) genes by driving their transcription (Bolsius et al., 2021). As PER and CRY proteins accumulate in the cytoplasm during a subjective day, the two heterodimers suppress the transcription mediated by BMAL1-CLOCK and ultimately inhibit the transcription of themselves (PER and CRY) (Daut and Fonken, 2019). This inhibition is relieved due to the ubiquitin-mediated degradation of those proteins. This time-delay cycle by transcription and accumulation/degradation of proteins transforms a negative-feedback homeostat into a self-sustained oscillator (approximately 24 h) (Gelder, 1998; Bolsius et al., 2021). Other regulatory genes, such as retinoic acid *Receptor–related Orphan Receptors* (RORs) and the REV-ERB proteins *Nuclear Receptor subfamily 1, group D, member 1 or 2* (NR1D1 and NR1D2), activate or inhibit the transcription of BMAL1 respectively. RORα competes with NR1D1 in binding to the BMAL1 and induces its expression while NR1D1 represses its transcription (Huang et al., 2011).

The individual difference in chronotypes may be associated with the clock genes, although we still do not know the potential physiological or molecular mechanisms. So far, there is much evidence that clock gene polymorphisms are related to the chronotype, which includes *Aryl Hydrocarbon Receptor Nuclear Translocator-Like Gene* (ARNTL), CLOCK, PER, CRY, *F-Box protein* (FBXL), *Regulator of G-protein Signaling* (RGS), *Adenylate kinase* (AK), NR1D1, 2, *D-site of albumin promoter Binding Protein* (DBP), *Basic Helix–Loop–Helix family gene member E40* (BHLHE40, 41), *Timeless Gene* (TIM), *Nuclear Factor, Interleukin 3 regulated* (NFIL3), and RORC. A recent GWAS in 697,828 individuals identified 351 loci related to chronotype, including many essential SCN clock genes, such as PER1, PER2, PER3, CRY, FBXL3, and ARNTL (Jones et al., 2019). Similar results suggested that PER2, RGS16, FBXL13, and AK5 were related to chronotypes (Jones et al., 2016; Kalmbach et al., 2017). RGS16 regulates cAMP signaling by synchronizing intercellular communication between pacemaker neurons in the SCN (Jones et al., 2016). In a GWAS on a larger population, NR1D2 rs4131403 was found to be related to chronotype (Maukonen et al., 2020). NFIL3 rs2482705 and RORC rs3828057 were inversely associated with evening chronotype (Kripke et al., 2014).

Furthermore, these polymorphisms of clock genes are also related to psychiatric disorders. Compared to healthy controls, the brain issues of participants with MDD inhibited abnormal circadian gene expression, including ARNTL, PER1-3, NR1D1, DBP, and BHLHE40, 41 (Li et al., 2013). In addition, genetic mechanisms of chronotype can influence seasonality in MDD and BD in an age-related manner (Ferrer et al., 2020). CRY1 was also correlated to MDD, ADHD (attention deficit hyperactivity disorder), insomnia, and anxiety (Onat et al., 2020). Women with the PER3 genotype have fewer depressive symptoms and more anxiety symptoms. Importantly, there is no association between chronotype and PER3 polymorphism (Silva A. et al., 2020). This conclusion may arise because the gender-specific effects of chronotype and depression were overlooked (Weiss et al., 2020). Other scholars also identified PER3 mutations associated with chronotype and anxiety (Liberman et al., 2017). Suzuki et al. (2017) tested BD patients and found that CLOCK rs1801260, associated with evening chronotype, is related to the discrepancy between subjective and objective severity of depressive symptoms. Similarly, the variant of the CLOCK gene showed an association with the efficacy of antidepressants in MDD patients (Ma et al., 2019). Moreover, ARNTL rs7107287 was related to depressive and stress symptoms and the negative impact of seasonality; TIM rs10876890 was related to hyperthymic temperament; and TIM rs2291738 was related to chronotype, which indicated that there were no associations between chronotype and affective dysfunction (Jankowski and Dmitrzak-Weglarz, 2017).

Other genes that were not the clock were also related to chronotype and psychiatric disorders. A study found that a candidate for Serotonin 2A receptor gene (5HT2A) receptor (HTR2A), which is located on chromosome 13q14-q21, was associated with mental (depression, schizophrenia) and behavioral (suicidal and aggressive behaviors) disorders and was associated with chronotype in a healthy population (Yeom et al., 2020). Additionally, GWAS of the UK Biobank also indicated HTR6 rs2050122 was related to chronotype. Some clock genes related to chronotype may modulate the association between chronotype and psychiatric disorders.

In summary, these polymorphisms of clock genes constituted the circadian oscillators are associated with chronotype and psychiatric disorders. However, the molecular and physiological mechanisms are unclear, particularly in how clock gene polymorphisms contribute to symptoms or characteristics of those disorders.

### Brian characteristics: Structure and function

The structural and functional differences of the brain for chronotype may be the direct reason for mental health issues. Regarding the brain structure, compared to eveningness, morning chronotype is related to lower regional gray matter density in the precuneus, the left posterior parietal cortex, and their adjacent areas, and to higher regional gray matter density in the bilateral orbitofrontal cortex and the hypothalamic areas around the bilateral SCN. Moreover, the results suggested a relationship between morning chronotype and lower cognitive functions, higher competence in pro-social and self-disciplinary behavior, and less attentional and emotional problems in brain structure level (Takeuchi et al., 2015). A structural magnetic resonance imaging study indicated that although chronotype is not related to total hippocampal volume, there is an association between evening chronotype and localized atrophy in the mid-anterior region of the right hippocampus independent of age, gender, sleep quality, and mood, which was explained to prolonged exposure to glucocorticoids and stress (Horne and Norbury, 2018a). A recent MRI study suggested that the evening chronotype is associated with a greater volume of the left anterior occipital sulcus (Evans et al., 2021). This difference from the visual cortex may reflect the effect of chronotype on the input signal of the environment to the circadian system. A diffusion tensor imaging study suggested the existence of differences in white matter integrity in the frontal and temporal lobes, cingulate gyrus, and corpus callosum in the evening chronotype compared to the morning and intermediate chronotype (Rosenberg et al., 2014). The chronotype-specificity whiter matter characteristics in corpus callosum may be related to BDs, while frontal lobes play essential roles in substance use disorders.

The default mode network (DMN), which consists of the posterior cingulate cortex (PCC), precuneus, medial prefrontal cortex (mPFC), inferior parietal lobule (IPL), and bilateral temporal cortex (BTC), is vital in maintaining consciousness awakening, emotion processing, attentional control, and working memory. Regarding brain function, many studies reported the association between mental disorders and altered functional connectivity of the DMN. A study on the brain’s resting-state functional connectivity suggested that functional connectivity of the DMN is fundamentally different in evening and morning chronotype individuals during working days (Facer-Childs et al., 2019a). Tian et al. (2020) observed that those evening chronotype individuals with poor sleep quality had higher precuneus-mPFC connectivity, which fully mediated the effect of chronotype on sleep quality. In addition, the evening chronotypes exhibited less mPFC reactivity during reward anticipation and more ventral striatum reactivity during win outcomes than the morning chronotype, demonstrating reduced regulatory control and elevated reward sensitivity, which may contribute to alcohol abuse (Hasler et al., 2013). Indeed, mood disorders like depression and anxiety are commonly comorbid with substance abuse, which the common neurobiological process may explain. Compared with morning chronotypes, evening chronotypes showed a diurnal variation of positive affect in line with the diurnal activity of the affective neural circuitry (mPFC and striatum), potentially constituting a risk for mood disorders among evening chronotype individuals (Hasler et al., 2012). The evening chronotype is associated with increased sensitivity to negative emotional facial expressions in the bilateral amygdala and reduced dorsal anterior cingulate cortex (dACC)–amygdala functional connectivity, suggesting that the evening chronotype is related to impaired emotional regulation circuitry (Horne and Norbury, 2018b). On the other hand, specific circadian rhythm changes have been identified in the nucleus accumbens (NAcc) in a depression-like mice model induced by chronic stress (Logan et al., 2015). The clock genes of MDD patients had an abnormal expression in the dorsolateral prefrontal cortex (DLPFC), anterior cingulate cortex (ACG), hippocampus, amygdala, NAcc, and cerebellum (Li et al., 2013).

Taken together, a growing body of evidence suggested the neural difference between chronotypes on the level of structure (gray matter, white matter, visual cortex, and hippocampal) and function (DMN, reward circuits, and limbic system), which contributes to the differences in psychological performance and etiology of mental disorders.

### Dual role of neuroendocrinology

Neurotransmitter switching may provide a core biological basis for chronotypes and chronotherapeutics (Wirz-Justice and Benedetti, 2020). Under normal circumstances, the onset of melatonin secretion occurs about 2 h before bedtime; core body temperature is the lowest overnight (2–3 h before waking); plasma cortisol concentrations reach their lowest point in the evening and peak shortly after waking in the morning (arousal). The temporal association between those physical parameters’ circadian rhythms and sleep timing is different (phase advanced, phase delayed, and internal desynchrony) (Hickie and Rogers, 2011). Melatonin, a pineal neurohormone, is activated by the circadian clock locked in the SCN and limited by light exposure and secretion following a 24-h cycle (inhibited by day and activated at night). Melatonin receptors are widely distributed in the brain, including in the SCN of the hypothalamus, substantia nigra, hippocampus, cerebellum ventral tegmental area, and the NAcc (Hickie and Rogers, 2011). Researchers have found specific differences in melatonin rhythms in individuals of different chronotypes. That is, those with evening chronotype have later melatonin rhythms with decreased mean levels and peaks (Lack et al., 2009; Razavi et al., 2019; McHill et al., 2021). Dim-light melatonin onset, which is a circadian phase marker, varies by almost two hours between chronotypes (Taillard et al., 2011) and shows chronotype-dependent effects on weekly and seasonal variation (both the DLMO and sleep time advanced one hour in summer than winter and delayed on weekdays compared to on weekends) (Zerbini et al., 2021).

In a sleep deprivation study, attenuated salivary melatonin secretion was identified in female MDD patients (Birchler-Pedross et al., 2011). Melatonin and other hormones may also play a role in the relationship between evening chronotype and obesity (Sun et al., 2020). In a sample of breast cancer patients, a negative correlation was found between melatonin levels and insomnia severity and depression severity (Zaki et al., 2020). Indeed, some neurotransmitters (dopamine, noradrenaline, serotonin, and tryptophan) involved in the etiology of psychiatric disorders participate directly in melatonin synthesis (Adan et al., 2012). The serotonergic system is associated with mood and circadian rhythm, and it seems to serve as a mediator between childhood trauma, circadian disruption, and mood disorder (Park, 2020). Serotonin plays adaptive roles in circadian rhythm, mood, anxiety, and cognitive functions (Bacque-Cazenave et al., 2020). Due to the bidirectional communication between serotonin and circadian rhythm and the influence of stress-induced changes in cortisol, the serotonergic system plays an essential role in intermediating the circadian regulation of vulnerability to MDD (Daut and Fonken, 2019). In terms of treatment, the evening chronotype reported poorer efficacy of SSRI medications (McGlashan et al., 2018). Cortisol, an endogenous hormone, has a circadian change and is regulated by negative feedback loops within the hypothalamic-pituitary-adrenal (HPA) axis (Girshkin et al., 2016). The disrupted HPA axis function is associated with psychiatric disorders such as MMD, BD, and schizophrenia (Wong et al., 2000; Menke, 2019). In healthy adolescents and adults, the morning chronotype showed higher levels of cortisol awakening response than the evening chronotype (Randler and Schaal, 2010; Correa et al., 2014). It seems that the earlier the chronotype, the higher the level of cortisol secretion (Minelli et al., 2021). However, some other researchers have found the opposite result of no significant difference in chronotype and cortisol levels when investigated in small and limited samples (Dockray and Steptoe, 2011; Toda et al., 2013).

### Different age stages show various features in chronotypes

The age-related changes in chronotype exist throughout the life span after controlling for demographic and socioeconomic factors. Age is a significant predictor of chronotype (Randler and Bilger, 2009; Roenneberg et al., 2019). Chronotype strongly depends on age (Holler et al., 2021), and it is well-known that the elderly are more likely to wake up and sleep early. In general, morning chronotype is predominant during babyhood (Simpkin et al., 2014). The evening chronotype is more prevalent among young adults (ages 25–34), while the morning chronotype has a greater proportion in the middle and old-age populations (Merikanto and Partonen, 2020). According to the MCTQ database from Roenneberg et al. (2004) children are early chronotypes, and the distribution of later chronotypes increases with the age of groups increasing from 10 to 20. After 20, chronotypes tend to be early (phase advance) until 70 (Roenneberg et al., 2004). Notably, results about age and chronotype measured by MEQ should be carefully considered since the inappropriate classification of the original version may not be suitable for the middle-aged population (Taillard et al., 2004). On the other hand, cohort effects or other confounders are needed since most research is cross-sectional.

The age-related intrinsic circadian period may explain the association between chronotype and age. The phase and amplitude of melatonin, core body temperature, and cortisol vary at different age stages. The intrinsic circadian period is greater (more than 24 h) (Carskadon et al., 1999). The phase of melatonin rhythm shows a similar trend among different age groups (Logan and McClung, 2019). Apart from melatonin, the sensitivity of zeitgeber may differ in various ages. For instance, deterioration of the eyes may weaken the effect of the light/dark cycle (Hood and Amir, 2017). In addition, based on the same data, the tendency of females to shift to a later chronotype seems to occur earlier than it does for boys around the age of 20, which indicates a gender effect in chronotype (Roenneberg et al., 2004).

### Gender differences cannot be ignored

Gender differences exist between psychiatric disorders and chronotype, respectively. Psychiatric disorders are characterized by marked gender differences. For example, females are more likely to develop MDD and show more severe symptoms, worse social function, and a higher risk of anxiety (Labonte et al., 2017; Hasbi et al., 2020). Although many studies have attempted to explore sex-specific effects on psychiatric disorders from various perspectives, such as socio-cultural factors (Salk et al., 2017), gene organization and expression (Labonte et al., 2017; Hasbi et al., 2020), neuro-function or neuro-immunity (Bekhbat and Neigh, 2018), the mechanisms remain unclear. However, chronotype is also characterized by sexual differences. Many studies exhibited that the morning chronotype represents a larger proportion among females, and the evening chronotype is more common among males (Tonetti et al., 2011; Adan et al., 2012; Borisenkov et al., 2012). However, the others suggested an opposite conclusion (Merikanto et al., 2012), or that there are no gender differences in the chronotypes (Zimmermann, 2011; Walsh et al., 2021). Duarte et al. (2014) investigated 14,650 volunteers and demonstrated that, compared to males, females were more morning-oriented before 30 years old while were more evening-oriented after the age of 45 years, and found no gender differences between the ages of 30–45 years. This result showed the gender differences in the plasticity of the circadian timekeeping system in men and women throughout ontogeny (Duarte et al., 2014). Furthermore, there are gender differences linking the chronotype and psychiatric disorders. A study of more than 5,000 non-shift-working adults indicated that evening chronotype was related to depression only in women, not men. The evening chronotype among females has some unfavorable aspects concerning school and work in young and old females (Fabbian et al., 2016). A potential explanation is the gender difference in melatonin, sex hormones, and behavioral and psychological factors (sleep quality, alcohol intake) (Kim et al., 2020). In addition, the circadian gene PER3 variable number of tandem repeats was independently related to chronotype only in males (Weiss et al., 2020). Taken together, both the chronotype and psychiatric disorders showed significant sex-specific effects, although the causes and mechanisms are unclear.

### The light/dark cycle is the primary external zeitgeber

The light/dark cycle is the primary zeitgeber synchronizing circadian rhythm in a wide array of species (Czeisler et al., 2005) and is significantly related to circadian rhythmicity by regulating SCN (Jagannath et al., 2017; Wirz-Justice et al., 2021). The visual effects of light rely on photoreceptor cells (rod and cones) which coordinate the image-forming tasks and lead to vision. On the other hand, the non-visual light effects depend on intrinsically photosensitive retinal ganglion cells (ipRGCs) to regulate physiological function, behavior, and emotion, such as circadian rhythm, the pupillary light reflexes, sleep, and mood (Guido et al., 2020). The ipRGCs are a subset of retinal ganglion cells containing melanopsin (Opn4), a member of the G-protein coupled receptor family (Diaz et al., 2016). The light/dark cycle synchronizes circadian rhythm depending on the time and duration of daily light exposure, the intensity, and the spectral properties (Touitou et al., 2016). Light exposure generally advances the circadian phase in the morning, while in the evening, it delays it. The phase response curve (PRC) reflects the relationship between the time light exposure and phase shift of circadian rhythm (Minors et al., 1991). There are large differences in daily light exposure between chronotypes: the morning chronotypes have more minutes of daily bright light exposure (> 1000 lux) than the evening chronotypes (Goulet et al., 2007). Moreover, after a four-hour exposure to blue light, EEG results showed that individuals with morning chronotype had increased theta and low alpha spectral power in the afternoon compared to those exposed to polychromatic white light, indicating that morning chronotype individuals may be more sensitive to light (Siemiginowska and Iskra-Golec, 2020).

Artificial light weakens the strength of the natural light/dark cycle (depending on the difference between daytime and nighttime light intensity) and delays the circadian phase in almost all chronotypes except the definite morning chronotype (Roenneberg et al., 2003, 2019; Roenneberg and Merrow, 2016). Among adolescents, those living in an environment with more outdoor light at night have a stronger evening chronotype orientation than those not (Vollmer et al., 2012). Light exposure at night will disrupt circadian rhythm and lead to adverse consequences for human health (Bedrosian and Nelson, 2017). The animal model indicated that an SCN-independent pathway, which provides excitatory synaptic input to the neurons of the peri-habenular nucleus (PHb) of the dorsal thalamus through ipRGCs, mediated the regulation of mood (Fernandez et al., 2018). The higher correlated color temperature of light exposure is conducive to stimulating a more positive mood during daytime (Xiao et al., 2021). In addition, consecutive and acute night light exposure induced depression-like behavior in mice (Walker et al., 2020).

On the other hand, the light therapies based on a theory of circadian rhythm and sleep homeostasis are effective in depressive disorders. Particularly, bright light therapy is highly effective in both SAD and non-seasonal depression (Maruani and Geoffroy, 2019) and is more effective than dim red light in non-seasonal unipolar depression patients with evening chronotype (Chan J. W. et al., 2020).

### Social factors with increasing importance in modern society

In humans, social factors (such as shift work, alarm clocks, and regular eating) are one of the most important zeitgebers (only next to the light/dark cycle). The use of artificial light from electronic devices makes it possible for activity at night, which will also impact our normal circadian rhythm.

Shift work is a special working arrangement in which holiday, night, or other non-mainstream working times feature. Paramedics, firefighters, and police are occupations generally involved in shift work. Notably, it is important to examine chronotypes and the types of shift work separately when researching the influence of shift work on health. Juda et al. (2013) did elaborate work on 238 shift workers and indicated that the morning chronotypes have shorter sleep duration, higher SJL, and higher levels of sleep disturbance during night shifts (10 PM to 6 AM). On the contrary, the evening chronotypes show a similar pattern on morning shifts (6 AM to 2 PM). In addition, on evening-shift days, the evening chronotype has a longer sleep duration and better sleep quality than the morning chronotypes, while the reverse is true for morning-shift days (Juda et al., 2013). In other words, compared to the morning chronotypes, evening subjects are more adapted to night shifts while morning subjects are similar to morning shifts. This adaptability may be because the active/rest cycle of chronotypes is consistent with different shift schedules separately. However, there are various types of shift work in the real world. For instance, a rotating shift is a schedule of 2 days of morning duty followed by two days of night duty with four days of break among the rosters (Khan et al., 2020). The discrepancy between the shift schedule and chronotypes, or circadian misalignment, contributes to shift workers having more severe insomnia, excessive daytime sleepiness, and worse mood disturbances, such as depression and anxiety. A meta-analysis of longitudinal studies indicated that shift work is associated with worse mental health, specifically depressive symptoms. This conclusion is consistent with another meta-analysis showing that night shift work is related to an increased risk of depression (Lee et al., 2017; Torquati et al., 2019). The night shift workers are exposed to long-term artificial light at night, which may lead to chronic desynchronization of their circadian rhythm. In addition, a systematic investigation also demonstrated that female nurses with evening chronotype are more prone to anxiety, insomnia, and fatigue (Lopez-Soto et al., 2019). The study collected data from 10637 participants and demonstrated that shift workers with evening chronotype were more vulnerable to mood disorders manifesting as sleep problems or susceptibility to artificial light exposure (Cheng et al., 2021). A study of paramedics in Australia indicated that individuals with evening chronotype have worse mental health, poorer sleep quality, and lower well-being than those with morning chronotype (Khan et al., 2020). Evening chronotype was also associated with depressive moods in nurses and firefighters (Choi et al., 2020). Taken together, these above findings may demonstrate that, despite different chronotypes being more adapted to corresponding shift works, the evening chronotype showed worse adaptability in variably real-world shift works.

The relationship between excessive use of electronic devices and evening chronotype was confirmed in children, adolescents, and adults (Fossum et al., 2014; Eid et al., 2020; Przepiorka et al., 2021). Evening chronotype individuals also showed more frequent use of electronic devices than morning chronotypes (Akçay et al., 2021). The evening subjects are more novelty-seeking and open-minded than the morning (Adan et al., 2012). These personalities may be why they spend more time on electronic devices than the morning chronotypes, and the same for insomnia. Electronic devices in bed were correlated with both evening chronotype and insomnia (Fossum et al., 2014). In other words, those people with evening chronotype or insomnia have to use electronic devices to pass the time. On the other hand, the information from electronic devices (such as games, videos, and social media) is very attractive and may cause a later sleep time and reduced sleep duration by promoting physiological and psychological arousal (Cain and Gradisar, 2010). After controlling for chronotype, the relationship between electronic device use and shorter sleep duration and longer sleep latency remains but is weakened (Hisler et al., 2020). In addition, artificial light, mainly blue light, may impact the normal circadian rhythm and contribute to a phase delay of the circadian clock (Touitou et al., 2016). The non-visual effects of light may explain the association between electronic use and evening chronotype. Light from self-luminous devices may suppress melatonin and delay the sleep phase due. In particular, adolescents showed more sensitivity to light (Figueiro and Overington, 2016). Since the majority of studies are cross-sectional, it remains unclear whether the evening chronotypes use electronic devices because they need to pass time until feeling sleepy, or the light from these devices and the physiological and psychological arousal from the contents lead to a phase delay of circadian rhythm. Alternatively, these processes could work together in the real world. Nonetheless, the evening chronotypes who overuse electronic devices will accumulate sleep debt every workday, leading to SJL, which contributes to psychiatric disorders. Indeed, individuals with evening chronotype and frequent internet use were found to be more depressive (Przepiorka et al., 2021). Furthermore, we cannot ignore the influence of network information. A study of a massive data set of Twitter content in the United Kingdom indicated that positive words were more likely to be used during the day. In contrast, negative words (indicating sadness and anger) appeared at night more frequently (Dzogang et al., 2017).

### Psychological and behavioral mechanism

Personality is a relatively stable predisposition and affects patterns of thinking, feeling, and acting (Friedman and Kern, 2014). The characteristics of personality in different chronotypes are important for understanding the relationship between chronotype and psychiatric disorders. According to the review by Adan et al. (2012), who summarized research evidence, the morning chronotypes are more conscientious, persistent, introverted, agreeable, and emotionally stable, while the evening chronotypes are more extraverted, impulsive, novelty-seeking, open-minded, and more likely to have psychoticism and a less adaptive emotional profile. Later researchers showed a similar conclusion. A meta-analysis investigated the relationship between chronotype and the Big Five factors of personality and indicated that evening chronotype is associated with extraversion and open-mindedness, while morning chronotype is strongly and positively related to conscientiousness and has a unique and negative association with neuroticism (Lipnevich et al., 2017). Similarly, a study composed of 1134 adolescents (age 10–14) demonstrated a large proportion of left-thinkers (prefer verbal and analytic strategies in processing information) among the morning chronotypes, while right-thinkers (more creative and intuitive) were among the evening chronotypes (Diaz-Morales and Escribano, 2013). Current evidence indicates that three personality traits (neuroticism, extraversion, and conscientiousness) are associated with psychiatric disorders such as depression and anxiety disorders. Higher neuroticism and lower extraversion predict a poorer course and response to treatment in depression. In addition, there is a strong negative relationship between conscientiousness and depression (Klein et al., 2011). Overall, the relationship between morning chronotype and neuroticism (negative) and conscientiousness (positive) may explain why morning chronotype is a protective factor of some mental disorders.

The emotion regulation strategy is critical in explaining the relationship between chronotype and psychiatric disorders. The association between evening chronotype and depression is mediated by a lower behavioral activation system (BAS) and lower positive affect (PA), and the evening chronotype is related to reduced reward responsiveness (Hasler et al., 2010). Diurnal variation exists in positive and negative affect, respectively (Dzogang et al., 2017), and the energetic arousal characteristic for chronotypes (Stolarski et al., 2016). After controlling for confounders, the evening chronotype showed impaired emotion regulation strategies (reduced cognitive reappraisal and increased expressive suppression), potentially increasing vulnerability to psychiatric disorders (Watts and Norbury, 2017). Researchers also found that reduced cognitive reappraisal and self-blame were associated with evening chronotype (Van den Berg et al., 2018). Increased expressive suppression was also correlated with poorer sleep quality (Latif et al., 2019). Evening chronotype was positively associated with perceived stress (You et al., 2020) and less use of the emotion regulation strategy, described as “putting [things] into perspective” (Henrich et al., 2021). A study of medical students indicated that evening chronotype individuals are less happy than others. This relationship between chronotype and happiness was significant after controlling for confounding variables, such as age and gender (Tan et al., 2020). Rumination is passive and repetitive thinking about negative emotions and their possible causes and consequences. It includes forced thinking and introspective thinking. Rumination was thought to be a unique cognitive predisposing factor of depression and was associated with evening chronotype by mediating the relationship between chronotype and depression (Antypa et al., 2017) and potentially aggravating the association between depression and cognition (Sun et al., 2014). A functional connectivity study observed that the evening chronotype was correlated with the precuneus-mPFC functional connectivity involved in self-referential processing (Tian et al., 2020). Rumination is also related to self-referential processing in evening chronotype and depression (Antypa et al., 2017), demonstrating a neural mechanism of the association between chronotype and mental diseases. Social support provided by family, friends, and teammates seems to be a potential mechanism by which social support is a partial mediator between evening chronotype and depressive symptoms (Wills et al., 2021). A recent study concluded that morning chronotypes have higher social support and mindfulness (Walsh et al., 2021). Compared to other chronotypes, evening chronotypes have conflicts in their social lives, potentially leading to low support and feelings of loneliness. One possible reason is that evening chronotypes may have sufficient social support but may be unaware or unwilling to recognize their support because of negative cognitive bias (Walsh et al., 2021). Overall, evening chronotype is highly linked with negative emotion strategies (such as reward responsiveness, cognitive reappraisal, and expressive suppression), rumination, and negative cognitive bias toward social support, which contributes to psychiatric disorders.

Cognitive functions reflect the brain’s characteristics, states, and interrelations of objective things, including sensation, memory, perception, attention, and execution. Executive function is the ability to flexibly integrate and coordinate different cognitive processes to achieve a specific goal and composes working memory, inhibition control, and cognitive flexibility. There is a synchrony effect of chronotype, showing better performance on cognitive function at their optimal times of day than at their non-optimal times (Hahn et al., 2012). In other words, the evening chronotypes do better in the evening while the morning chronotypes do better during the daytime. Previous research revealed that, despite the evening chronotypes showing higher intelligence and cognitive ability than the morning chronotypes, they have worse academic achievement (Preckel et al., 2011; Diaz-Morales and Escribano, 2013). The synchrony effect may explain it since the test time is not too late, usually at school. The evening chronotypes have higher correct answer rates than the morning chronotypes in the 2-back and 3-back tasks, which commonly test working memory. The synchrony effect of chronotype appears only at a higher complexity of the task (3-back) (Schmidt et al., 2015). However, current evidence is unable to clarify the relationship between chronotype and cognitive functions. The interplay between cognitive function and emotion regulation may be executed by the same set of neural mechanisms and may contribute to psychiatric disorders (Gruber and Cassoff, 2014). Cognitive impairments are not only symptoms of some psychiatric disorders but also the main factor of functional recovery. Evening chronotype and poor sleep quality contribute to cognitive impairments in MDD independently (Cabanel et al., 2019). Impaired response inhibition (considered a marker of vulnerability to BD and substance use disorders), high impulsivity, and disinhibition are more frequently associated with evening chronotype and maybe a core personality trait involved in circadian rhythm (Kang et al., 2015). In summary, the synchrony effect of chronotype on various cognition is confirmed. Evening chronotype seems to have vulnerabilities in cognition, which contributes to psychiatric disorders. Nonetheless, future works should prove these results since current evidence in cognitive tasks, experimental designs, samples, and measuring methods shows large differences.

### Sleep quality: Mediator or predictor of chronotype?

As we described already, it is clear that chronotype is somehow connected with sleep disorders. The chronotypes showed different sleep problems. Morning chronotype is related to sleep phase-advance syndrome and difficulty in maintaining sleep. Simultaneously, evening chronotype is associated with difficulty in initiating sleep and morning sleepiness. Even in those who are considered good sleepers, this relationship exists (morningness to sleep phase-advance syndrome and eveningness to morning sleepiness) (Taillard et al., 2001). Chronotype was the strongest predictor of sleep disorders (Sun et al., 2019). People with evening chronotype have worse sleep quality and more severe insomnia, while being a morning chronotype is a protective factor (Salfi et al., 2021). In addition, evening chronotype was an independent predictor of mental health issues, such as MDD, BD, and anxiety disorders, and was positively associated with depressive symptoms. A large population study in Japan suggested that evening chronotype is related to an increased risk of depressive symptoms after adjusting for sleep factors (Kitamura et al., 2010). Simor’s conclusion supported this hypothesis after adjusting for age, gender, circadian misalignment, and sleep complaints (Simor et al., 2015). Chan J. W. et al. (2014) suggested that evening chronotype is an independent risk factor of non-remission in MDD.

On the other hand, sleep disorders or sleep phenotypes, such as insomnia, hypersomnia, and daytime sleepiness, have proven to be independently linked to mental disorders, especially depressive disorders (Wang et al., 2019). Indeed, sleep disorders and mood disorders are risk factors for each other. Recent studies have demonstrated that sleep disorders are associated with depression and anxiety (Cox and Olatunji, 2019; Dagnew et al., 2020; Furihata et al., 2020). In a survey of 8580 subjects aged 16–74 years, 83% of depressed patients have reported insomnia symptoms, and 41% of them were diagnosed with insomnia depending on DSM-IV criteria (Stewart et al., 2006). Inadequate sleep was associated with depressive symptoms in a study of US and Chinese adolescents (Yang et al., 2020). The GWAS of over one million individuals revealed the causal effects of insomnia on depression (Jansen et al., 2019). A study of healthy older adults indicated that older people with insomnia had reduced motivation and sensitivity to monetary reward, suggesting that a dysregulated reward system may become a mechanism whereby insomnia contributes to late-life depression (Boyle et al., 2020). Sleep disturbance is associated with reduced well-being in college students (Fischer et al., 2020). Insomnia severity, especially difficulty in initiating sleep but not sleep duration, is a predictor of first onset MDD (Correa et al., 2014). A prospective study showed that sleep irregularity was associated with depressive episodes in BD patients (Ng et al., 2016).

A large body of evidence demonstrated that poor sleep quality or sleep disorders mediate the association between evening chronotype and depressive symptoms in healthy subjects and patients with mood disorders (Van den Berg et al., 2018; Caruso et al., 2020; Li et al., 2020; Tian et al., 2020; Uzer and Yucens, 2020b; Lin et al., 2021; Zhou et al., 2021) or other adverse outcomes (Cox and Olatunji, 2019; Kandeger et al., 2019; Najem et al., 2020; Bradford et al., 2021). A study of older adults with depression indicated that the relationship between evening chronotype and depression is fully mediated by insomnia severity (Uzer and Yucens, 2020b). Clinical evaluation of patients with BD indicated that poor sleep quality mediated the association between evening chronotype and residual depressive symptoms (Caruso et al., 2020). A recent study revealed that insomnia partially mediates the effect of chronotype on depression and anxiety, completely mediating the association between chronotype and suicidal ideation (Bradford et al., 2021). The relationship between evening chronotype and psychological distress showed a mediation effect (Hou et al., 2020). Zhou et al. (2021) suggested that resilience could moderate the effects of chronotype and sleep quality on depressive symptoms and that enhanced resilience could effectively alleviate the symptoms of mood disorders. Evening chronotype was associated with high levels of problematic use of social media, more psychological distress (such as depression, higher suicide ideation, and lower PA), and daytime sleepiness. In addition, sleep quality and severe insomnia were significant mediators in these associations (Lin et al., 2021). Depressive symptoms improved after insomnia was cured in evening chronotype adolescents (Chan N. Y. et al., 2020).

However, there are opposite outcomes. The mediated effect of sleep problems between chronotype and mood disorders is unproven. A large cohort study indicated that the association between evening chronotype and MMD could not be fully explained by sleep problems, such as insomnia and inadequate sleep duration (Antypa et al., 2016). According to mediation analysis, hopelessness, defined as negative expectations for the future and core symptoms of MDD, mediates the relationship between evening chronotype and depressive symptoms, but not sleep quality (Uzer and Yucens, 2020a). Taken together, although there were conflicting results regarding the relationship between psychiatric disorders, chronotype, and sleep disorders, the general view is that evening chronotype is an independent risk factor for psychiatric disorders, especially depressive disorders, in which sleep disorders show a partially or fully mediating effect.

## Association between chronotype and psychiatric disorders

### Chronotype and depressive disorders

Depressive disorders are characterized by significant and persistent depressive symptoms as the primary clinical feature, often accompanied by the rhythmical fluctuation of mood and high morbidity, heavy burden, and great social attention (Mrazek et al., 2014; Malhi and Mann, 2018). MDD represents the classic condition in this group of disorders. It is characterized by discrete episodes of at least two weeks (although most episodes last considerably longer) involving clear-cut changes in affect, cognition, neurovegetative functions, and inter-episode remissions (Arlington and Publishing, 2013). Although many hypotheses have tried to explain the pathogenesis of depressive disorders, such as the monoamine depletion hypothesis, the neuroplasticity impairment hypothesis, the neuroendocrine disruption hypothesis, and the circadian rhythm disruption hypothesis, the potential mechanism remains unclear (Malhi and Mann, 2018; Lynch et al., 2020). Depression often shows diurnal mood variation (Morris et al., 2009), diurnal changes in hormone levels (Hickie and Rogers, 2011), and sleep disorders, such as early awakening, insomnia, and shallow sleep (Germain and Kupfer, 2008; Emens et al., 2009). Disruption of circadian rhythmicity is associated with the severity of depressive symptoms, the impairment of cognitive function, and clinical treatment, efficacy, and prognosis. The evening chronotype is often referred to as a risk factor for depression disorder, whereas the morning chronotype is a protective factor (Adan et al., 2012; Chan J. W. et al., 2014).

Many studies have indicated that the evening chronotype is significantly associated with depressive symptoms in non-clinical samples. Most studies have found that evening chronotype is consistently associated with depression and is an independent risk factor (Simor et al., 2015). This conclusion was made in studies on children, adolescents, and adults (Gau et al., 2007; Alvaro et al., 2014; Chan N. Y. et al., 2020; Eid et al., 2020; Weiss et al., 2020; Koo et al., 2021) and in different countries (Alvaro et al., 2014; Kivela et al., 2018; Walsh et al., 2021). Even excluding some effects of social factors like shift work, evening chronotype was found to be related to a higher incidence of depression (investigated in 1165 non-shift workers by Taillard et al., 2001). A recent meta-analysis of 43 studies demonstrated a reliable association between evening chronotype and depressive symptoms (Norbury, 2021). A large population-based study of 10,503 adults indicated that evening chronotypes are at higher risk of having depressive disorders, needing to take antidepressant medication, and experiencing depressive symptoms (Merikanto et al., 2015).

In addition, compared to non-psychiatric controls, participants with a high risk of psychosis and patients with depressive disorders exhibited a greater preference for the evening chronotype (Lunsford-Avery et al., 2021). There was also an association between evening chronotype and suicidality, which is firmly and fully mediated by depressive symptoms (Mokros et al., 2021). A study of 236 homogenous samples of young men suggested that more severe depressive symptoms were associated with evening chronotype, higher perceived stress, poorer sleep quality, and lower levels of morning salivary cortisol (Tonon et al., 2020). Mendelian randomization analysis indicated a protective relationship between morning diurnal preference (correspond to a 1-h earlier sleep midpoint) and a lower risk of MDD (Daghlas et al., 2021). However, unlike most studies, an epidemiological survey found that morning chronotype subjects have shorter sleep duration and were more likely to be patients with depressive disorder (Lemoine et al., 2013). This result is probably due to the lack of healthy subjects, the use of rMEQ (only six questions), the high age (average age of 51.64 years), and the large gender ratio gap (29.3% were males) of depressive subjects.

The evening chronotype was independently related to depressive disorders (Chan J. W. et al., 2014; Antypa et al., 2016; Au and Reece, 2017; Ferrer et al., 2020; Lunsford-Avery et al., 2021). A meta-analysis of 36 studies demonstrated that the evening chronotype is related to mood symptoms, such as MDD, BD, and SAD (Au and Reece, 2017). A case-control study of young persons suggested higher rates of evening chronotype in young individuals with depression and anxiety than in healthy controls (Fares et al., 2015). The evening chronotype was associated with the severity of the disease and a higher occurrence of suicidal thoughts (Gaspar-Barba et al., 2009). In a 1-year naturalistic follow-up study of 253 MDD patients, evening chronotypes have more severe depressive symptoms, higher incidence of suicide, and more suicidal ideation (Chan J. W. et al., 2014). A literature review shows that the evening chronotype was a critical characteristic of suicidal ideation and behavior (Rumble et al., 2018). Depressive patients with evening chronotypes have higher levels of negative affect on weekends (Bruckmann et al., 2020). A 7-year follow-up longitudinal study showed that, first, the earlier chronotype individuals experience milder depressive symptoms, and second, the changes in chronotype are consistent with changes of the severity of depressive but not of anxiety symptoms over time (Druiven et al., 2020). A genetic study in a large population demonstrated that morning chronotype was negatively related to depressive symptoms and MDD while positively correlated with subjective well-being (Jones et al., 2019). In short, the evening chronotype is a risk factor for depression, whereas the morning chronotype seems to be a protective factor. The relationship between chronotype and depression (or depressive symptoms) was examined in studies that used different samples, such as healthy individuals (children, adolescents, and adults) and patients with MDD. This relationship has clinical implications for improving prevention, diagnosis, and treatment.

As we described above, the hypothesis of circadian dysfunction is that the genetic vulnerabilities, zeitgeber, age, and sex, work together to contribute to depression in the evening chronotypes. The mutations of clock genes could induce variation in the intrinsic period but also affect hormone secretion such as glucocorticoids (Shi et al., 2016) and serotonin (Daut and Fonken, 2019). Depression commonly emerges in adolescents or young adults (Crouse et al., 2021), while the evening chronotype has a higher distribution in similar ages. The characteristics of age and gender in chronotype are consistent with a depressed population, which may be the result or phenomenon of the underlying mechanism that we do not yet know. From a psychological view, the evening chronotypes tend to have psychoticism and a less adaptive emotion profile in personality, in addition to being more associated with negative emotion strategies, rumination, and cognitive bias. These mental traits are potentially increasing vulnerability to depressive disorders. On the other hand, some researchers suggested that sleep disorders may contribute to mood disorders independently. However, evening chronotype is an independent risk factor for psychiatric disorders, especially depressive disorders, in which sleep disorders show a partially or fully mediating effect. Although it seems evident that evening chronotype is independently and positively related to depression as its risk factor, the physiopathology remains unclear. Nonetheless, bright light therapy is highly effective in treating depressive disorders, which suggests the hypothesis of circadian and sleep disruption show excellent prospects.

### Chronotype and bipolar disorders

Bipolar disorders, including BD-I and BD-II, are mood disorders. BD-I is characterized by at least one manic episode, which may have been preceded by and may be followed by hypomanic or major depressive episodes. BD II is characterized by a clinical course of recurring mood episodes consisting of one or more major depressive episodes and at least one episode of hypomania (Arlington and Publishing, 2013). Circadian rhythm disruption, evening chronotype, abnormal hormone secretion, and vulnerability of the CLOCK gene have been documented in BD (Takaesu, 2018; Ferrer et al., 2020; Ozdogan et al., 2020). The association between evening chronotype and BD was confirmed in subjective (questionnaire) and objective (actigraph) chronotype measurements (Gershon et al., 2018; Kaufmann et al., 2018). A recent large cross-sectional study of 773 BD patients and 146 control subjects demonstrated evening chronotype as a discrete clinical subtype in BD patients who were younger and had an earlier age of onset, worse clinical symptoms (more depressive and manic episodes, higher rate of suicide attempts), more comorbid anxiety, and substance use disorder (Romo-Nava et al., 2020a). An 18-month prospective study among 80 BD patients (the most prevalent diagnosis was BD-I, *n* = 69) demonstrated that the evening chronotype was associated with more severe anxiety, more mood episodes, and being overweight, suggesting a poor prognosis for BD (Melo et al., 2020). Evening chronotype was relevant in increased depression, mania, and insomnia in patients with BD, and lithium is more effective in treating patients with morning chronotype (McCarthy et al., 2019). Simultaneously, a meta-analysis showed similar findings suggesting that chronotype may be a potential biomarker of lithium treatment in BD patients even though only five studies were included (Xu et al., 2021). For the comorbidity aspect, compared to other types of BD, individuals with evening chronotype have increased rates of anxiety, substance use disorders (Romo-Nava et al., 2020a), and disordered eating behavior (or more unhealthy dietary patterns) (Romo-Nava et al., 2020b). A prospective and naturalistic follow-up study indicated that sleep irregularity might be a risk for the onset of major depressive episodes in BD (Ng et al., 2016). Patients with BD have been reported to have less social rhythm regularity than healthy individuals, a factor potentially predicting affective episodes (Shen et al., 2008). Dysfunction of circadian rhythms provides a new and hopeful perspective for treating BD patients at the genetic level (Oliveira et al., 2018). However, disrupted circadian rhythm and evening chronotype are common in BD, but their influence on mood episodes is unclear (Melo et al., 2017).

Notably, other studies have discovered no significant association between chronotypes and the two BD subtypes (Lewis et al., 2020). This case-control study of 4,672 BD participants and 5,714 control subjects suggested that BD-I had a greater genetic liability to longer sleep duration as did BD-II to insomnia. Nevertheless, the variables of age and education, which show significant influence on chronotype, were not adjusted due to there being no control participants. In short, evening chronotype may be a high-risk subtype in BD because of the worse symptoms and more comorbidity, while current evidence is weak and shows unclear mechanisms. Genetics may be the most important prospect because of high hereditary susceptibility. Future studies need to focus on the relationship between chronotype and subtypes of BD.

### Chronotype and anxiety disorders

Anxiety disorders include disorders that share the features of excessive fear, anxiety, and related behavioral disturbances. Fear is the emotional response to a real or perceived imminent threat, whereas anxiety is the anticipation of a future threat (Arlington and Publishing, 2013). The association between chronotype and anxiety has been found in many studies, and chronotype may be one of the contributors to anxiety processes (Cox and Olatunji, 2019). Some studies have shown a relationship between a high level of anxiety symptoms and morning chronotype (Haraden et al., 2019). Others indicated that anxiety is associated with evening chronotype (Gau et al., 2007; Passos et al., 2017; Walsh et al., 2021), and individuals with anxiety showed higher rates of evening chronotype than healthy individuals did (Fares et al., 2015; Walsh et al., 2021). A study with 3,160 healthy participants suggests that the evening chronotype shows more severe symptoms of anxiety, depression, and sleep disorders than other chronotypes (Walsh et al., 2021). A study of 64 patients with chronic primary insomnia indicated that the evening chronotype is related to higher trait, state, and pre-sleep state anxiety, while the proportion of female subjects was too high (*n* = 50) (Passos et al., 2017). In addition, other studies on university students concluded that the evening preference was related to poorer sleep quality, which can be explained by higher sleep latency, shorter sleep time, late onset of sleep, and higher trait and state anxiety than students with a morning preference (Silva V. M. et al., 2020). The possible explanation is that sleep factor is more related to anxiety symptoms than chronotype.

In fibromyalgia patients, the evening chronotype was found to be related to increased severity of anxiety symptoms and poorer sleep quality (Turkoglu and Selvi, 2020). Extreme sleep duration is associated with elevated anxiety symptoms (Zhou et al., 2020). However, other research has demonstrated that chronotype is not associated with anxiety. In a longitudinal study, a chronotype change was not relevant to severity of anxiety symptoms (Druiven et al., 2020). Alvaro et al. (2014) suggested that the evening chronotype during adolescence uniquely predicted depression and insomnia but not anxiety disorders. Considering the current evidence, although there is an exact link between chronotype and anxiety symptoms in a non-clinical population, the underlying mechanism and causality remain unclear. In particular, there are few studies of samples with clinically diagnosed anxiety disorders.

### Chronotype and schizophrenia

Schizophrenia is a multifactorial disorder defined by abnormalities in one or more of five domains, namely delusions, hallucinations, disorganized thinking or speech, grossly disorganized, or abnormal motor behavior, and negative symptoms (Arlington and Publishing, 2013). Circadian rhythm disruption may be a risk factor for schizophrenia by affecting the disorder onset and severity of symptoms (Delorme et al., 2020). Some previous reviews have suggested little evidence linking chronotype and schizophrenia (Ahn et al., 2008; Kivela et al., 2018; Taylor and Hasler, 2018). However, with the deepening of research interest in circadian rhythmicity in recent years, more and more evidence has emerged. A recent meta-analysis of five studies comprising more than 300 patients and over 600 healthy controls indicated that patients with schizophrenia are more evening-oriented than healthy individuals, independent of age, gender, and the type of questionnaire assessing chronotype (Linke and Jankowski, 2021). Genome-wide association studies (GWAS) demonstrated that the chronotype is associated with schizophrenia (Lane et al., 2016) and that people with the morning chronotype have a lower risk of schizophrenia and greater subjective well-being (Jones et al., 2019). A case-control study indicated that patients with schizophrenia are more prone to have evening chronotype than healthy participants are, but this correlation was not clarified (Kilicaslan, 2020). In summary, current evidence could not provide a clear relationship between chronotype and schizophrenia. More clinical case-control and longitudinal studies are needed in the future.

### Chronotype and substance use disorders

Substance use disorders are defined as the repeated use of one or more psychoactive substance to the extent that the user is periodically or chronically poisoned (Arlington and Publishing, 2013). Disrupted circadian rhythmicity may contribute to addiction or substance abuse. Growing evidence suggests that the evening chronotype is associated with substance abuse, especially alcohol and tobacco (FernÁNdez-Mendoza et al., 2010; Hasler et al., 2017; Borisenkov et al., 2018; Taylor et al., 2020). A research study involving 1,271 individuals in a Spanish university found that individuals with evening chronotype were frequent users of stimulants (chocolate, tobacco, cocaine, amphetamines, and ecstasy) and depressants (alcohol and cannabis) (FernÁNdez-Mendoza et al., 2010). A longitudinal study of adolescents showed that evening chronotype prospectively predicted increased alcohol and marijuana abuse (Hasler et al., 2017). Similarly, college students with evening chronotype reported a higher level of alcohol use disorders. However, the author failed to test the mediated effect of emotion regulation in chronotype and alcohol use disorder (Taylor et al., 2020). Alcohol abuse among evening chronotypes may be accompanied by altered neural responses to the reward circuit (Hasler et al., 2013). Notably, all this research above investigated non-clinical populations. Nonetheless, Kervran et al. (2015) collected and analyzed 333 subjects with an addiction who enrolled as outpatients and assessed them by clinical interview. The results demonstrated that evening chronotype is significantly related to cannabis addiction, non-substance addiction, poly-problematic addiction, and mood disorders, while not to the severity of addiction due to the lack of difference in severity scores in these clinical samples (Kervran et al., 2015). Food addiction (FA) is a concept whereby a preference for high-calorie or highly processed foods eventually leads to changes in behavior and physiology. Evening chronotype and depressive symptoms were associated with FA (Borisenkov et al., 2020). However, a cross-sectional study of 1,323 university students indicated that evening chronotype was indirectly related to FA, which is mediated by insomnia and impulsivity (Kandeger et al., 2019). Internet addiction was associated with evening chronotype, a trait that may foster internet addiction (Randler et al., 2013). The clock–specific therapies may improve the outcomes when treating substance use disorders (Logan et al., 2014; Gulick and Gamsby, 2018). In this regard, compared to the healthy population, a higher trend in morning chronotype was observed in patients with substance use disorders who were under treatment for more than three months (Antunez et al., 2016). It is helpful to improve circadian rhythm in the prevention and therapy of substance use disorders patients (Capella et al., 2018). Overall, the evening chronotype seems to be a risk factor in the onset and maintenance of substance use disorders, while the morning chronotype is considered a protection factor (Adan, 2012).

Disruption of circadian rhythms is rarely considered in the etiology of substance use disorders, although it may increase susceptibility to addiction. Similar to depressive disorders, prevalence of substance use disorders and chronotype show parallel traits. In other words, evening chronotype orientation and the prevalence are highest among adolescents and young adults (Gulick and Gamsby, 2018). This result may be due to the development of the prefrontal cortex and reward circuit, and patients with substance use disorders show aberrant expression of clock genes in reward regions (Falcon and McClung, 2009). As we described above, the evening chronotypes inhibited altered neural response to reward-related areas (mPFC and ventral striatum) compared to the morning chronotypes, which may lead to alcohol abuse (Hasler et al., 2013).

### Chronotype and eating disorders

Eating disorders, which include rumination disorder, avoidant/restrictive food intake disorder, anorexia nervosa, bulimia nervosa, and binge eating disorder, are characterized by a persistent disturbance of eating or eating-related behavior resulting in altered consumption or absorption of food, significantly impairing physical health or psychosocial functioning (Arlington and Publishing, 2013). Evening chronotype was related to eating disorders, and evidence was found in children (Rodriguez-Cortes et al., 2022), adolescents (Fleig and Randler, 2009; Rodriguez-Cortes et al., 2022), adults (Kandeger et al., 2018; Sun et al., 2020), and BD patients (Romo-Nava et al., 2020b). A recent meta-analysis demonstrated that children and adolescents with an evening chronotype had a higher body mass index, consumed more junk food, or were more predisposed to suffer from FA and night eating syndrome (Rodriguez-Cortes et al., 2022). A scoping review demonstrated that evening chronotype was related to later mealtimes, breakfast skipping habits, higher energy intake, notably before bedtime, and an unhealthy diet structure (Mazri et al., 2019). Individuals with binge eating and night eating behavior were more likely to be evening chronotypes than morning chronotypes. In a study that included 1197 middle-aged men and women (mean age 48.2 years), the evening chronotype was found to be independently associated with obesity, and the potential mechanism of this association was influenced by unhealthy dietary habits or eating behavior disorders (Sun et al., 2020). A recent cohort study of US women indicated that evening chronotype contributed to a poorer habitual diet, partially mediating the association between evening chronotype and poorer cardiovascular health (Zuraikat et al., 2021). Gender differences could also be seen in eating behavior disorders. A cross-sectional study of 400 students from a Lebanese university suggested that females are more likely to engage in emotional eating than males, and males with lower chronotype scores are more prone to uncontrolled eating (Aoun et al., 2019). In general, there is a significant association between evening chronotype and eating disorders, though most research focused on binge eating disorders. In addition, gender differences are evident. The association between evening chronotype and eating behavior may be influenced by differences in personal worldviews regarding time perspectives (Guenther and Stolarski, 2019) or food craving traits (Teixeira et al., 2020). The personality of morning chronotypes, such as conscientiousness and persistence, may protect them from unhealthy diet habits. Moreover, irregular eating behaviors may delay the circadian phase and contribute to evening chronotype orientation.

## Conclusion and discussion

The stability of circadian rhythm is an essential prerequisite for human health, and chronotype as a stable state of people has been associated with physical and psychiatric disorders. The review of recent research indicates that chronotype plays a vital role in depressive disorders, BD, anxiety disorders, substance use disorders, and eating disorders. The findings are as follows.

(1) Depression may be the most closely related condition to chronotype: the evening chronotype is a risk factor for depressive disorders, whereas the morning chronotype is a protective factor.
(2) Evening chronotype individuals with BD show more severe symptoms and comorbidity. Chronotype may be a potential biomarker of lithium treatment in BD.
(3) The evening chronotype is only related to anxiety symptoms due to the lack of evidence on research samples diagnosed with anxiety disorders.
(4) The association between chronotype and schizophrenia remains unclear, although new evidence has emerged.
(5) Evening chronotype individuals reported a higher rate of substance use disorders. The evening chronotype seems to be a risk factor in the onset and maintenance of substance use disorders, while the morning chronotype is considered a protective factor. It is beneficial for patients with substance use disorders to use chronotherapy to establish regular daily activities.
(6) The evening chronotype is significantly associated with eating disorders, though most studies focused on binge eating disorders.

Most importantly, although psychiatric disorders are classified depending on their particular syndrome, most patients in the real world have at least one comorbidity (such as depression, anxiety, and sleep disorders). This comorbidity is evident because some psychiatric disorders share common genetic and neural physiological processes. In summary, chronotype is controlled by genetics and zeitgebers, while it is deeply affected by age and gender. Two main oscillators regulate chronotype: the circadian systems and the homeostatic process. The polymorphisms of clock genes control the intrinsic circadian period and directly/indirectly affect the sleep process. After the expression of clock genes, the execution of related functions depends on the structure and function of the brain. In humans, the light/dark cycle mainly entrains circadian oscillator compared with social factors. Some studies in blind people supported this conclusion and demonstrated that blind patients show more disrupted circadian rhythms though they live with strong social activities (Sack et al., 2000; Golombek and Rosenstein, 2010), especially in those without light perception (Hartley et al., 2018).

The evening chronotype accumulates sleep debt and leads to SJL or circadian misalignment due to their different phases from zeitgeists (such as artificial light at night and social activities) with the development of industrialization. In other words, the conflict between modern social lifestyle and personal preference in sleep time may partly explain why chronotype is a risk factor for some psychiatric disorders. Chronotype represents a stable state but not a trait (Roenneberg et al., 2019). Chronotype changes under different entraining environments (Facer-Childs et al., 2019b), while it will relapse without intervention since its genetic basis determines the intrinsic circadian period. This nature of chronotype reflects the interaction between innate factors and environmental factors.

**FIGURE 1:**
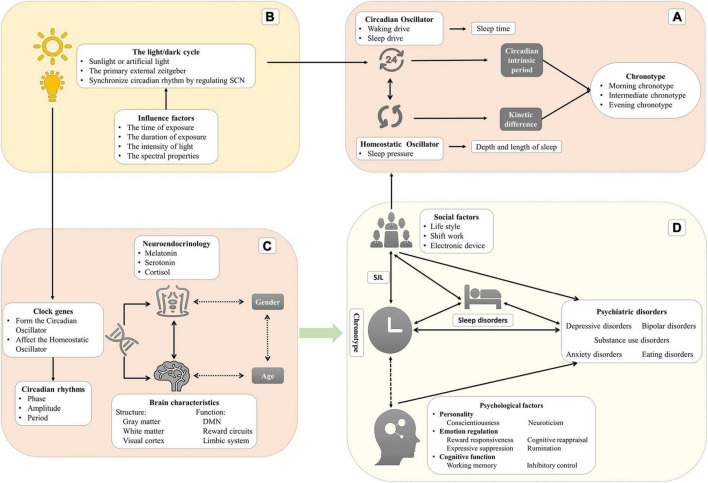
Putative potential mechanisms between chronotype and psychiatric disorders. **(A)** The two oscillators regulate sleep parameters and lead to chronotypes. **(B)** The light/dark cycle and its influence factors. **(C)** The physiological bases contribute to differences in chronotypes. **(D)** The potential mechanisms and relationship between chronotype and psychiatric disorders. The direction of the arrows indicates the possible causality. The solid and dotted lines correspond to the strength of the connection. SCN, suprachiasmatic nuclei; DMN, default mode network; SJL, social jet lag.

However, as the pathogenesis of most psychiatric disorders remains unclear, we could not clarify the mechanism and causal relationship between chronotype and psychiatric disorders. Current hypotheses stress the effect of aberrant neural circuit neuroendocrinology in the etiology of mental disorders. As described above, the two extreme chronotypes exhibit significant differences in gray and white matters at the structural level and their functional regions, such as DMN and rewarding circuits. Furthermore, the process of neuroendocrinology is deeply involved in circadian and homeostatic systems and participates in the etiology of psychiatric disorders. Together with neural development and hormone secretion, there are age-related changes and gender differences in both chronotype and psychiatric disorders. In addition, psychology and behavior play a significant role. The evening chronotypes are more likely to demonstrate negative personalities and poor emotion regulation than the morning chronotype. The evening chronotypes are less conscientious, less persistent, more impulsive, and more likely to have negative cognitive bias and rumination, which leads to worse performances despite their better intelligence and cognitive functions. In addition, these psychological and behavioral differences depend on the variation of the brain (Takeuchi et al., 2015). To our knowledge, what is notable is that sleep disorders share a similar physiological process with chronotype and are comorbid with many psychiatric disorders. In our opinion, sleep disorders show a mediating effect between chronotype and other mental disorders (Figure 1).

There are some limitations to the current research. First, most studies are cross-sectional. Therefore, there is a need for longitudinal and experimental research. Due to limitations in the study’s design, the causality between chronotype and psychiatric disorders is unclear. Little evidence was found on the neuroimaging and psychological mechanisms of chronotypes, notably concerning psychiatric disorders. Further investigation and improvement would be worthwhile. There is a need for an experimental design to examine the relationship between chronotype and mood disorders, in which chronotype is not feasibly manipulated or randomly assigned (Au and Reece, 2017). In a cortisol-induced depression-like behavior mice model, alterations in circadian rhythm preceded the onset of depression, demonstrating that circadian rhythm may cause or predict the onset of depression (Spulber et al., 2015). Moreover, a large population of GWAS demonstrated no evidence to suggest that poor mental health influences chronotype, providing a reference from a genetic perspective (Jones et al., 2019). Second, most studies used the MEQ or MCTQ to assess and classify chronotypes, but the self-reported data may have been biased. Few studies have objectively employed physiological measures of chronotype, such as wrist accelerometry, body temperature, and hormone measurement (Kivela et al., 2018), because objective measures are not feasible within a large population sample (Evans et al., 2021). In particular, there is a discrepancy between subjective and objective measures, which may obstruct the experimental results (Chung et al., 2020). The third is the variability of chronotypes. Aging, gender, and intervention can change chronotype, requiring future research to investigate these confounders. Fourth, some studies relied on non-clinical or student samples. Therefore, their findings were not applicable to clinical populations or other age groups.

Evening chronotype affects early diagnosis, treatment, and prognosis. Since MDD (Gau et al., 2007; Gaspar-Barba et al., 2009) and BD (Romo-Nava et al., 2020a) patients with evening chronotype have a high risk of suicide, it is vital not only to investigate the association between chronotype and suicide in psychiatric disorders but also to prevent, and to intervene early in, the onset of suicidal ideation. The development of precision medicine means that chronotype identification has a potential advantage in developing targeted and personalized treatments for psychiatric disorders, especially mood disorders (Adan et al., 2012; Gershon et al., 2018; Lynch et al., 2020). The effects of treatment of circadian dysfunction in psychiatric disorders with psychosocial, pharmacological, and light therapy are encouraging (Gold and Kinrys, 2019). Chronotype could also be a potential biomarker for lithium treatment in patients with BD (Xu et al., 2021). Some drugs that affect circadian rhythms, such as melatonin and its analogs, can potentially treat neuropsychiatric disorders (Hickie and Rogers, 2011).

## Author contributions

HWZ wrote the original text, drew the Figure 1 and Table 1, and revised and assisted with editing the manuscript. HLZ helped generate ideas for the framework of the manuscript, conducted the linguistic sorting investigation, and assisted with editing the manuscript. RY provided editorial direction and assisted with revising the manuscript. ZY helped generate ideas for the framework of the manuscript and revised the manuscript. QL helped generate ideas for the framework of the manuscript and revised the manuscript. All authors contributed to and approved the final manuscript.

**TABLE 1:** Questionnaires for chronotype.

Name	Questions	Variables	Evaluation and Classification	
MEQ	19 items	• Sleep and wake-up times	Definitely morning type	70–86
		• Preferred times for physical and Mental activity	Moderately morning type	59–69
		• Subjective alertness	Intermediate type	42–58
			Moderately evening type	31–41
			Definitely evening type	16–30
MCTQ	29 items	**(workdays and work-free days)**	**MSFsc**	
		• So_*w*_/SO_*f*_	Extreme early type	
		• GU_*w*_/GU_*f*_	Moderate early type	
		• SD_*w*_/SD_*f*_	Slight early type	
		• TBT_*w*_/TBT_*f*_	Normal type	
		• MSW/MSF	Slight late type	
			Moderate late type	
			Extreme late type	
CSM	13 items	• Activity planning	Morning Type	44-55
		• Morning alertness	Intermediate Type	23–43
		• Evening alertness	Evening Type	13–2 2

SO, sleep onset; GU, local time of getting out of bed; SD, sleep duration; TBT, total time in bed; MS, mid-sleep. MSFsc, the mid-sleep on work-free days corrected for “oversleep” due to the sleep debt accumulated during the workdays, calculation: If SD_f_ ≤ SD_w_, MSFsc = MSF or If SD_f_ > SD_w_, MSFsc = MSF - (SD_f_ - SD_w_)/2. The categorization of chronotype by using MCTQ is based on quantile of the MSFsc scores in samples.

## References

[B1] Adan A. (2012). A chronobiological approach to addiction. *J. Subst. Use* 18 171–183. 10.3109/14659891.2011.632060

[B2] Adan A. Almirall H. (1991). Horne and Östberg morningness–eveningness questionnaire: a reduced scale. *Person. Indiv. Diff.* 1991 241–253. 10.1016/0191-8869(91)90110-W

[B3] Adan A. Archer S. N. Hidalgo M. P. Milia L. Di Natale V. Randler C. (2012). Circadian typology: a comprehensive review. *Chronobiol. Int.* 29 1153–1175. 10.3109/07420528.2012.719971 23004349

[B4] Ahn Y. M. Chang J. Joo Y. H. Kim S. C. Lee K. Y. Kim Y. S. (2008). Chronotype distribution in bipolar I disorder and schizophrenia in a Korean sample. *Bipolar Disord.* 10 271–275. 10.1111/j.1399-5618.2007.00573.x 18271906

[B5] Akçay B. D. Akçay D. Yetkin S. (2021). The effects of mobile electronic devices use on the sleep states of university students. *Alpha Psychiatry* 22 31–37. 10.5455/apd.99831 PMC9590654 36426202

[B6] Alvaro P. K. Roberts R. M. Harris J. K. (2014). The independent relationships between insomnia, depression, subtypes of anxiety, and chronotype during adolescence. *Sleep Med.* 15 934–941. 10.1016/j.sleep.2014.03.019 24958244

[B7] Antunez J. M. Capella M. D. Navarro J. F. Adan A. (2016). Circadian rhythmicity in substance use disorder male patients with and without comorbid depression under ambulatory and therapeutic community treatment. *Chronobiol. Int.* 33 1410–1421. 10.1080/07420528.2016.1223092 27611843

[B8] Antypa N. Verkuil B. Molendijk M. Schoevers R. Penninx B. Van Der Does W. (2017). Associations between chronotypes and psychological vulnerability factors of depression. *Chronobiol. Int.* 34 1125–1135. 10.1080/07420528.2017.1345932 28759270

[B9] Antypa N. Vogelzangs N. Meesters Y. Schoevers R. Penninx B. W. (2016). Chronotype associations with depression and anxiety disorders in a large cohort study. *Depress Anxiety* 33 75–83. 10.1002/da.22422 26367018

[B10] Aoun C. Nassar L. Soumi S. El Osta N. Papazian T. Rabbaa Khabbaz L. (2019). The cognitive, behavioral, and emotional aspects of eating habits and association with impulsivity, chronotype, anxiety, and depression: a cross-sectional study. *Front. Behav. Neurosci.* 13:204. 10.3389/fnbeh.2019.00204 31555108 PMC6742717

[B11] Arlington V. Publishing U. A. P. (2013). *Diagnostic and statistical manual of mental disorders, 5th edition (DSM-5).* Washington, D.C: American Psychiatric Association.

[B12] Aschoff J. Pohl H. (1978). Phase relations between a circadian rhythm and its zeitgeber within the range of entrainment. *Naturwissenschaften* 65 80–84. 10.1007/BF00440545 345129

[B13] Au J. Reece J. (2017). The relationship between chronotype and depressive symptoms: a meta-analysis. *J. Affect. Disord.* 218 93–104. 10.1016/j.jad.2017.04.021 28463712

[B14] Bacque-Cazenave J. Bharatiya R. Barriere G. Delbecque J. P. Bouguiyoud N. Di Giovanni G. (2020). Serotonin in animal cognition and behavior. *Int. J. Mol. Sci.* 21:5. 10.3390/ijms21051649 32121267 PMC7084567

[B15] Baehr E. K. Revelle W. Eastman C. I. (2000). Individual differences in the phase and amplitude of the human circadian temperature rhythm: with an emphasis on morningness-eveningness. *J. Sleep Res.* 9 117–127. 10.1046/j.1365-2869.2000.00196.x 10849238

[B16] Bauducco S. Richardson C. Gradisar M. (2020). Chronotype, circadian rhythms and mood. *Curr. Opin. Psychol.* 34 77–83. 10.1016/j.copsyc.2019.09.002 31786494

[B17] Bedrosian T. A. Nelson R. J. (2017). Timing of light exposure affects mood and brain circuits. *Transl. Psychiatry* 7:e1017. 10.1038/tp.2016.262 28140399 PMC5299389

[B18] Bekhbat M. Neigh G. N. (2018). Sex differences in the neuro-immune consequences of stress: Focus on depression and anxiety. *Brain Behav. Immun.* 67 1–12. 10.1016/j.bbi.2017.02.006 28216088 PMC5559342

[B19] Birchler-Pedross A. Frey S. Chellappa S. L. Gotz T. Brunner P. Knoblauch V. (2011). Higher frontal EEG synchronization in young women with major depression: a marker for increased homeostatic sleep pressure?. *Sleep* 34 1699–1706. 10.5665/sleep.1440 22131608 PMC3208848

[B20] Bolsius Y. G. Zurbriggen M. D. Kim J. K. Kas M. J. Meerlo P. Aton S. J. (2021). The role of clock genes in sleep, stress and memory. *Biochem. Pharmacol.* 191:114493. 10.1016/j.bcp.2021.114493 33647263 PMC9487905

[B21] Borisenkov M. F. Kosova A. L. Kasyanova O. N. (2012). Impact of perinatal photoperiod on the chronotype of 11- to 18-year-olds in northern European Russia. *Chronobiol. Int.* 29 305–310. 10.3109/07420528.2011.653612 22390243

[B22] Borisenkov M. F. Polugrudov A. S. Paderin N. M. Bakutova L. A. (2018). Young inhabitants of the North with late chronotype and social jetlag consume more high-calorie foods and alcohol. *Biolog. Rhyth. Res.* 50 418–428. 10.1080/09291016.2018.1455867

[B23] Borisenkov M. F. Popov S. V. Tserne T. A. Bakutova L. A. Pecherkina A. A. Dorogina O. I. (2020). Food addiction and symptoms of depression among inhabitants of the european north of russia: associations with sleep characteristics and photoperiod. *Eur. Eat. Disord. Rev.* 28 332–342. 10.1002/erv.2728 32153116

[B24] Borisenkov M. F. Vetosheva V. I. Kuznetsova Y. S. Khodyrev G. N. Shikhova A. V. Popov S. V. (2019). Chronotype, social jetlag, and time perspective. *Chronobiol. Int.* 36 1772–1781. 10.1080/07420528.2019.1683858 31658823

[B25] Boyle C. C. Cho J. H. Eisenberger N. I. Olmstead R. E. Piber D. Sadeghi N. (2020). Motivation and sensitivity to monetary reward in late-life insomnia: moderating role of sex and the inflammatory marker CRP. *Neuropsychopharmacology* 45 1664–1671. 10.1038/s41386-020-0735-7 32544926 PMC7419294

[B26] Bradford D. R. R. Biello S. M. Russell K. (2021). Insomnia symptoms mediate the association between eveningness and suicidal ideation, defeat, entrapment, and psychological distress in students. *Chronobiol. Int.* 2021 1–12. 10.1080/07420528.2021.1931274 34100311

[B27] Bruckmann K. F. Hennig J. Muller M. J. Fockenberg S. Schmidt A. M. Cabanel N. (2020). Influence of chronotype on daily mood fluctuations: pilot study in patients with depression. *BJPsych. Open* 6:e17. 10.1192/bjo.2019.103 32019630 PMC7176862

[B28] Cabanel N. Schmidt A. M. Fockenberg S. Bruckmann K. F. Haag A. Muller M. J. (2019). Evening preference and poor sleep independently affect attentional-executive functions in patients with depression. *Psychiatry Res.* 281:112533. 10.1016/j.psychres.2019.112533 31521842

[B29] Cain N. Gradisar M. (2010). Electronic media use and sleep in school-aged children and adolescents: a review. *Sleep Med.* 11 735–742. 10.1016/j.sleep.2010.02.006 20673649

[B30] Capella M. D. M. Martinez-Nicolas A. Adan A. (2018). Circadian rhythmic characteristics in men with substance use disorder under treatment. influence of age of onset of substance use and duration of abstinence. *Front. Psychiatry* 9:373. 10.3389/fpsyt.2018.00373 30174624 PMC6107843

[B31] Carney C. E. Buysse D. J. Ancoli-Israel S. Edinger J. D. Krystal A. D. Lichstein K. L. (2012). The consensus sleep diary: standardizing prospective sleep self-monitoring. *Sleep* 35 287–302. 10.5665/sleep.1642 22294820 PMC3250369

[B32] Carskadon M. A. Labyak S. E. Acebo C. Seifer R. (1999). Intrinsic circadian period of adolescent humans measured in conditions of forced desynchrony. *Neurosci. Lett.* 260 129–132. 10.1016/s0304-3940(98)00971-9 10025716

[B33] Caruso D. Meyrel M. Krane-Gartiser K. Benard V. Benizri C. Brochard H. (2020). Eveningness and poor sleep quality contribute to depressive residual symptoms and behavioral inhibition in patients with bipolar disorder. *Chronobiol. Int.* 37 101–110. 10.1080/07420528.2019.1685533 31690130

[B34] Cespedes Feliciano E. M. Rifas-Shiman S. L. Quante M. Redline S. Oken E. Taveras E. M. (2019). Chronotype, social jet lag, and cardiometabolic risk factors in early adolescence. *JAMA Pediatr.* 2019:3089. 10.1001/jamapediatrics.2019.3089 31524936 PMC6749538

[B35] Chan J. W. Lam S. P. Li S. X. Chau S. W. Chan S. Y. Chan N. Y. (2020). Adjunctive bright light treatment with gradual advance in unipolar major depressive disorder with evening chronotype - A randomized controlled trial. *Psychol. Med.* 2020 1–10. 10.1017/S0033291720003232 32924897

[B36] Chan J. W. Lam S. P. Li S. X. Yu M. W. Chan N. Y. Zhang J. (2014). Eveningness and insomnia: independent risk factors of nonremission in major depressive disorder. *Sleep* 37 911–917. 10.5665/sleep.3658 24790269 PMC3985112

[B37] Chan N. Y. Zhang J. Tsang C. C. Li A. M. Chan J. W. Y. Wing Y. K. (2020). The associations of insomnia symptoms and chronotype with daytime sleepiness, mood symptoms and suicide risk in adolescents. *Sleep Med.* 74 124–131. 10.1016/j.sleep.2020.05.035 32853897

[B38] Cheng W. J. Puttonen S. Vanttola P. Koskinen A. Kivimaki M. Harma M. (2021). Association of shift work with mood disorders and sleep problems according to chronotype: a 17-year cohort study. *Chronobiol. Int.* 38 518–525. 10.1080/07420528.2021.1885431 33588657

[B39] Choi S. J. Song P. Suh S. Joo E. Y. Lee S. I. (2020). Insomnia symptoms and mood disturbances in shift workers with different chronotypes and working schedules. *J. Clin. Neurol.* 16 108–115. 10.3988/jcn.2020.16.1.108 31942766 PMC6974840

[B40] Chung K. F. Poon Y. P. Y. Ng T. K. Kan C. K. (2020). Subjective-Objective Sleep Discrepancy in Schizophrenia. *Behav. Sleep Med.* 18 653–667. 10.1080/15402002.2019.1656077 31426678

[B41] Correa A. Molina E. Sanabria D. (2014). Effects of chronotype and time of day on the vigilance decrement during simulated driving. *Accid. Anal. Prev.* 67 113–118. 10.1016/j.aap.2014.02.020 24636873

[B42] Cox R. C. Olatunji B. O. (2019). Differential associations between chronotype, anxiety, and negative affect: a structural equation modeling approach. *J. Affect. Disord* 257 321–330. 10.1016/j.jad.2019.07.012 31302521 PMC6711779

[B43] Croce P. Quercia A. Costa S. Zappasodi F. (2018). Circadian rhythms in fractal features of eeg signals. *Front. Physiol.* 9:1567. 10.3389/fphys.2018.01567 30483146 PMC6240683

[B44] Crouse J. J. Carpenter J. S. Song Y. J. C. Hockey S. J. Naismith S. L. Grunstein R. R. (2021). Circadian rhythm sleep–wake disturbances and depression in young people: implications for prevention and early intervention. *Lancet Psychiatry* 8 813–823. 10.1016/s2215-0366(21)00034-1 34419186

[B45] Czeisler C. A. Buxton O. M. Sbs K. (2005). The human circadian timing system and sleep-wake regulation. *Prin. Pract. Sleep Med.* 375:94.

[B46] Daghlas I. Lane J. Saxena R. Vetter C. J. J. P. (2021). Genetically proxied diurnal preference, sleep timing, and risk of major depressive disorder. *JAMA Psychiatry* 78 903–910. 10.1001/jamapsychiatry.2021.0959 34037671 PMC8156187

[B47] Dagnew B. Andualem Z. Dagne H. (2020). Excessive daytime sleepiness and its predictors among medical and health science students of University of Gondar, Northwest Ethiopia: institution-based cross-sectional study. *Health Qual. Life Outcomes* 18:299. 10.1186/s12955-020-01553-3 32891148 PMC7487924

[B48] Daut R. A. Fonken L. K. (2019). Circadian regulation of depression: a role for serotonin. *Front. Neuroendocrinol.* 54:100746. 10.1016/j.yfrne.2019.04.003 31002895 PMC9826732

[B49] Delorme T. C. Srivastava L. K. Cermakian N. (2020). Are circadian disturbances a core pathophysiological component of schizophrenia?. *J. Biol. Rhythms* 35 325–339. 10.1177/0748730420929448 32498652

[B50] Diaz N. M. Morera L. P. Guido M. E. (2016). Melanopsin and the non-visual photochemistry in the inner retina of vertebrates. *Photochem. Photobiol.* 92 29–44. 10.1111/php.12545 26500165

[B51] Diaz-Morales J. F. Escribano C. (2013). Circadian preference and thinking styles: implications for school achievement. *Chronobiol. Int.* 30 1231–1239. 10.3109/07420528.2013.813854 24024592

[B52] Dijk D. J. von Schantz M. (2005). Timing and consolidation of human sleep, wakefulness, and performance by a symphony of oscillators. *J. Biol. Rhythms* 20 279–290. 10.1177/0748730405278292 16077148

[B53] Dockray S. Steptoe A. (2011). Chronotype and diurnal cortisol profile in working women: differences between work and leisure days. *Psychoneuroendocrinology* 36 649–655. 10.1016/j.psyneuen.2010.09.008 20950941

[B54] Druiven S. J. M. Hovenkamp-Hermelink J. H. M. Knapen S. E. Kamphuis J. Haarman B. C. M. Penninx B. (2020). Stability of chronotype over a 7-year follow-up period and its association with severity of depressive and anxiety symptoms. *Depress Anxiety* 37 466–474. 10.1002/da.22995 32065480 PMC7318352

[B55] Duarte L. L. Menna-Barreto L. Miguel M. A. Louzada F. Araujo J. Alam M. (2014). Chronotype ontogeny related to gender. *Braz. J. Med. Biol. Res.* 47 316–320. 10.1590/1414-431x20143001 24714814 PMC4075295

[B56] Duffy J. F. Rimmer D. W. Czeisler C. A. (2001). Association of intrinsic circadian period with morningness-eveningness, usual wake time, and circadian phase. *Behav. Neurosci.* 115 895–899. 10.1037//0735-7044.115.4.895 11508728

[B57] Dzogang F. Lightman S. Cristianini N. (2017). Circadian mood variations in Twitter content. *Brain Neurosci. Adv.* 1:2398212817744501. 10.1177/2398212817744501 29270466 PMC5736128

[B58] Eid B. Bou Saleh M. Melki I. Torbey P. H. Najem J. Saber M. (2020). Evaluation of chronotype among children and associations with bmi, sleep, anxiety, and depression. *Front. Neurol.* 11:416. 10.3389/fneur.2020.00416 32581995 PMC7291378

[B59] Emens J. Lewy A. Kinzie J. M. Arntz D. Rough J. (2009). Circadian misalignment in major depressive disorder. *Psychiatry Res.* 168 259–261. 10.1016/j.psychres.2009.04.009 19524304

[B60] Etain B. Milhiet V. Bellivier F. Leboyer M. (2011). Genetics of circadian rhythms and mood spectrum disorders. *Eur. Neuropsychopharmacol.* 21(Suppl. 4), S676–S682. 10.1016/j.euroneuro.2011.07.007 21835597

[B61] Evans S. L. Leocadio-Miguel M. A. Taporoski T. P. Gomez L. M. Horimoto A. Alkan E. (2021). Evening preference correlates with regional brain volumes in the anterior occipital lobe. *Chronobiol. Int.* 38 1135–1142. 10.1080/07420528.2021.1912077 33906520 PMC8243809

[B62] Fabbian F. Zucchi B. De Giorgi A. Tiseo R. Boari B. Salmi R. (2016). Chronotype, gender and general health. *Chronobiol. Int.* 33 863–882. 10.1080/07420528.2016.1176927 27148626

[B63] Facer-Childs E. R. Campos B. M. Middleton B. Skene D. J. Bagshaw A. P. (2019a). Circadian phenotype impacts the brain’s resting-state functional connectivity, attentional performance, and sleepiness. *Sleep* 42:5. 10.1093/sleep/zsz033 30763951 PMC6519915

[B64] Facer-Childs E. R. Middleton B. Skene D. J. Bagshaw A. P. (2019b). Resetting the late timing of ‘night owls’ has a positive impact on mental health and performance. *Sleep Med.* 60 236–247. 10.1016/j.sleep.2019.05.001 31202686

[B65] Falcon E. McClung C. A. (2009). A role for the circadian genes in drug addiction. *Neuropharmacology* 56(Suppl. 1), 91–96. 10.1016/j.neuropharm.2008.06.054 18644396 PMC2635341

[B66] Fares S. Hermens D. F. Naismith S. L. White D. Hickie I. B. Robillard R. (2015). Clinical correlates of chronotypes in young persons with mental disorders. *Chronobiol. Int.* 32 1183–1191. 10.3109/07420528.2015.1078346 26375049

[B67] Fernandez D. C. Fogerson P. M. Lazzerini Ospri L. Thomsen M. B. Layne R. M. Severin D. (2018). Light affects mood and learning through distinct retina-brain pathways. *Cell* 175 71–84e18. 10.1016/j.cell.2018.08.004 30173913 PMC6190605

[B68] FernÁNdez-Mendoza J. Ilioudi C. Montes M. I. Olavarrieta-Bernardino S. Aguirre-Berrocal A. De La Cruz-Troca J. J. (2010). Circadian preference, nighttime sleep and daytime functioning in young adulthood. *Sleep Biol. Rhythms* 8 52–62. 10.1111/j.1479-8425.2010.00430.x

[B69] Ferrer A. Costas J. Gratacos M. Martinez-Amoros E. Labad J. Soriano-Mas C. (2020). Clock gene polygenic risk score and seasonality in major depressive disorder and bipolar disorder. *Genes Brain Behav.* 19:e12683. 10.1111/gbb.12683 32573093

[B70] Figueiro M. Overington D. (2016). Self-luminous devices and melatonin suppression in adolescents. *Light. Res. Technol.* 48 966–975. 10.1177/1477153515584979

[B71] Fischer D. McHill A. W. Sano A. Picard R. W. Barger L. K. Czeisler C. A. (2020). Irregular sleep and event schedules are associated with poorer self-reported well-being in US college students. *Sleep* 43:6. 10.1093/sleep/zsz300 31837266 PMC7294408

[B72] Fleig D. Randler C. (2009). Association between chronotype and diet in adolescents based on food logs. *Eat Behav.* 10 115–118. 10.1016/j.eatbeh.2009.03.002 19447353

[B73] Fossum I. N. Nordnes L. T. Storemark S. S. Bjorvatn B. Pallesen S. (2014). The association between use of electronic media in bed before going to sleep and insomnia symptoms, daytime sleepiness, morningness, and chronotype. *Behav. Sleep Med.* 12 343–357. 10.1080/15402002.2013.819468 24156294

[B74] Foster R. G. Peirson S. N. Wulff K. Winnebeck E. Vetter C. Roenneberg T. (2013). Sleep and circadian rhythm disruption in social jetlag and mental illness. *Prog. Mol. Biol. Transl. Sci.* 119 325–346. 10.1016/B978-0-12-396971-2.00011-7 23899602

[B75] Friedman H. S. Kern M. L. (2014). Personality, well-being, and health. *Annu. Rev. Psychol.* 65 719–742. 10.1146/annurev-psych-010213-115123 24405364

[B76] Furihata R. Saitoh K. Suzuki M. Jike M. Kaneita Y. Ohida T. (2020). A composite measure of sleep health is associated with symptoms of depression among Japanese female hospital nurses. *Compr. Psychiatry* 97:152151. 10.1016/j.comppsych.2019.152151 31954287

[B77] Gaspar-Barba E. Calati R. Cruz-Fuentes C. S. Ontiveros-Uribe M. P. Natale V. De Ronchi D. (2009). Depressive symptomatology is influenced by chronotypes. *J. Affect Disord.* 119 100–106. 10.1016/j.jad.2009.02.021 19285347

[B78] Gau S. S. Shang C. Y. Merikangas K. R. Chiu Y. N. Soong W. T. Cheng A. T. (2007). Association between morningness-eveningness and behavioral/emotional problems among adolescents. *J. Biol. Rhythms* 22 268–274. 10.1177/0748730406298447 17517916

[B79] Gelder R. N. V. (1998). Circadian rhythms: eyes of the clock. *Curr. Biol.* 8 R798–R801. 9811593 10.1016/s0960-9822(07)00503-9

[B80] Gentry N. W. Ashbrook L. H. Fu Y. H. Ptacek L. J. (2021). Human circadian variations. *J Clin Invest* 131 16. 10.1172/JCI148282 34396981 PMC8363277

[B81] Germain A. Kupfer D. J. (2008). Circadian rhythm disturbances in depression. *Hum. Psychopharmacol.* 23 571–585. 10.1002/hup.964 18680211 PMC2612129

[B82] Gershon A. Kaufmann C. N. Depp C. A. Miller S. Do D. Zeitzer J. M. (2018). Subjective versus objective evening chronotypes in bipolar disorder. *J. Affect. Disord.* 225 342–349. 10.1016/j.jad.2017.08.055 28843917 PMC5626649

[B83] Girshkin L. O’Reilly N. Quide Y. Teroganova N. Rowland J. E. Schofield P. R. (2016). Diurnal cortisol variation and cortisol response to an MRI stressor in schizophrenia and bipolar disorder. *Psychoneuroendocrinology* 67 61–69. 10.1016/j.psyneuen.2016.01.021 26874562

[B84] Gold A. K. Kinrys G. (2019). Treating circadian rhythm disruption in bipolar disorder. *Curr. Psychiatry Rep.* 21:14. 10.1007/s11920-019-1001-8 30826893 PMC6812517

[B85] Golombek D. A. Rosenstein R. E. (2010). Physiology of circadian entrainment. *Physiol. Rev.* 90 1063–1102. 10.1152/physrev.00009.2009 20664079

[B86] Goulet G. Mongrain V. Desrosiers C. Paquet J. Dumont M. (2007). Daily light exposure in morning-type and evening-type individuals. *J. Biol. Rhythms* 22 151–158. 10.1177/0748730406297780 17440216

[B87] Gruber R. Cassoff J. (2014). The interplay between sleep and emotion regulation: conceptual framework empirical evidence and future directions. *Curr. Psychiatry Rep.* 16:500. 10.1007/s11920-014-0500-x 25200984

[B88] Guenther K. Stolarski M. (2019). Linking morningness with healthy eating behaviors: the mediating role of time perspectives. *Biol. Rhythm Res.* 52 1618–1630. 10.1080/09291016.2019.1684795

[B89] Guido M. E. Marchese N. A. Rios M. N. Morera L. P. Diaz N. M. Garbarino-Pico E. (2020). Non-visual opsins and novel photo-detectors in the vertebrate inner retina mediate light responses within the blue spectrum region. *Cell Mol. Neurobiol.* 2020:997. 10.1007/s10571-020-00997-x 33231827 PMC11441211

[B90] Gulick D. Gamsby J. J. (2018). Racing the clock: the role of circadian rhythmicity in addiction across the lifespan. *Pharmacol. Ther.* 188 124–139. 10.1016/j.pharmthera.2018.03.003 29551440

[B91] Hahn C. Cowell J. M. Wiprzycka U. J. Goldstein D. Ralph M. Hasher L. (2012). Circadian rhythms in executive function during the transition to adolescence: the effect of synchrony between chronotype and time of day. *Dev. Sci.* 15 408–416. 10.1111/j.1467-7687.2012.01137.x 22490180 PMC4103784

[B92] Haraden D. A. Mullin B. C. Hankin B. L. (2019). Internalizing symptoms and chronotype in youth: a longitudinal assessment of anxiety, depression and tripartite model. *Psychiatry Res.* 272 797–805. 10.1016/j.psychres.2018.12.117 30832201 PMC6498437

[B93] Hartley S. Dauvilliers Y. Quera-Salva M. A. (2018). Circadian rhythm disturbances in the blind. *Curr. Neurol. Neurosci. Rep.* 18:65. 10.1007/s11910-018-0876-9 30083814

[B94] Hasbi A. Nguyen T. Rahal H. Manduca J. D. Miksys S. Tyndale R. F. (2020). Sex difference in dopamine D1-D2 receptor complex expression and signaling affects depression- and anxiety-like behaviors. *Biol. Sex Differ.* 11:8. 10.1186/s13293-020-00285-9 32087746 PMC7035642

[B95] Hasler B. P. Allen J. J. Sbarra D. A. Bootzin R. R. Bernert R. A. (2010). Morningness-eveningness and depression: preliminary evidence for the role of the behavioral activation system and positive affect. *Psychiatry Res.* 176 166–173. 10.1016/j.psychres.2009.06.006 20132992 PMC2844473

[B96] Hasler B. P. Franzen P. L. de Zambotti M. Prouty D. Brown S. A. Tapert S. F. (2017). Eveningness and later sleep timing are associated with greater risk for alcohol and marijuana use in adolescence: initial findings from the national consortium on alcohol and neurodevelopment in adolescence study. *Alcohol. Clin. Exp. Res.* 41 1154–1165. 10.1111/acer.13401 28421617 PMC5488322

[B97] Hasler B. P. Germain A. Nofzinger E. A. Kupfer D. J. Krafty R. T. Rothenberger S. D. (2012). Chronotype and diurnal patterns of positive affect and affective neural circuitry in primary insomnia. *J. Sleep Res.* 21 515–526. 10.1111/j.1365-2869.2012.01002.x 22369504 PMC3371278

[B98] Hasler B. P. Sitnick S. L. Shaw D. S. Forbes E. E. (2013). An altered neural response to reward may contribute to alcohol problems among late adolescents with an evening chronotype. *Psychiatry Res.* 214 357–364. 10.1016/j.pscychresns.2013.08.005 24144507 PMC3852171

[B99] Henderson S. E. M. Brady E. M. Robertson N. (2019). Associations between social jetlag and mental health in young people: a systematic review. *Chronobiol. Int.* 36 1316–1333. 10.1080/07420528.2019.1636813 31387413

[B100] Henrich L. C. Antypa N. Van den Berg J. F. (2021). Sleep quality in students: associations with psychological and lifestyle factors. *Curr. Psychol.* 2021:9. 10.1007/s12144-021-01801-9

[B101] Hickie I. B. Rogers N. L. (2011). Novel melatonin-based therapies: potential advances in the treatment of major depression. *Lancet* 378 621–631. 10.1016/s0140-6736(11)60095-0 21596429

[B102] Hisler G. Twenge J. M. Krizan Z. (2020). Associations between screen time and short sleep duration among adolescents varies by media type: evidence from a cohort study. *Sleep Med.* 66 92–102. 10.1016/j.sleep.2019.08.007 31838456

[B103] Holler Y. Gudjonsdottir B. E. Valgeirsdottir S. K. Heimisson G. T. (2021). The effect of age and chronotype on seasonality, sleep problems, and mood. *Psychiatry Res.* 297:113722. 10.1016/j.psychres.2021.113722 33476898

[B104] Hood S. Amir S. (2017). The aging clock: circadian rhythms and later life. *J. Clin. Invest.* 127 437–446. 10.1172/JCI90328 28145903 PMC5272178

[B105] Horne C. M. Norbury R. (2018b). Late chronotype is associated with enhanced amygdala reactivity and reduced fronto-limbic functional connectivity to fearful versus happy facial expressions. *Neuroimage* 171 355–363. 10.1016/j.neuroimage.2018.01.025 29339309

[B106] Horne C. M. Norbury R. (2018a). Exploring the effect of chronotype on hippocampal volume and shape: a combined approach. *Chronobiol. Int.* 35 1027–1033. 10.1080/07420528.2018.1455056 29621410

[B107] Horne J. Ostberg O. J. I. J. O. C. (1976). A self-assessment questionnaire to determine morningness-eveningness in human circadian rhythms. *Int. J. Chronobiol.* 4 97–110. 1027738

[B108] Hou T. Zhang F. Mao X. Deng G. (2020). Chronotype and psychological distress among Chinese rural population: a moderated mediation model of sleep quality and age. *PLoS One* 15:e0241301. 10.1371/journal.pone.0241301 33125424 PMC7598484

[B109] Huang W. Ramsey K. M. Marcheva B. Bass J. (2011). Circadian rhythms, sleep, and metabolism. *J. Clin. Invest.* 121 2133–2141. 10.1172/JCI46043 21633182 PMC3104765

[B110] Islam Z. Hu H. Akter S. Kuwahara K. Kochi T. Eguchi M. (2020). Social jetlag is associated with an increased likelihood of having depressive symptoms among the Japanese working population: the furukawa nutrition and health study. *Sleep* 43:1. 10.1093/sleep/zsz204 31555821 PMC6985924

[B111] Jagannath A. Taylor L. Wakaf Z. Vasudevan S. R. Foster R. G. (2017). The genetics of circadian rhythms, sleep and health. *Hum. Mol. Genet.* 26 R128–R138. 10.1093/hmg/ddx240 28977444 PMC5886477

[B112] Jankowski K. S. Dmitrzak-Weglarz M. (2017). ARNTL, CLOCK and PER3 polymorphisms - links with chronotype and affective dimensions. *Chronobiol. Int.* 34 1105–1113. 10.1080/07420528.2017.1343341 28708003

[B113] Jansen P. R. Watanabe K. Stringer S. Skene N. Bryois J. Hammerschlag A. R. (2019). Genome-wide analysis of insomnia in 1,331,010 individuals identifies new risk loci and functional pathways. *Nat. Genet.* 51 394–403. 10.1038/s41588-018-0333-3 30804565

[B114] Jones S. E. Lane J. M. Wood A. R. van Hees V. T. Tyrrell J. Beaumont R. N. (2019). Genome-wide association analyses of chronotype in 697,828 individuals provides insights into circadian rhythms. *Nat. Commun.* 10:343. 10.1038/s41467-018-08259-7 30696823 PMC6351539

[B115] Jones S. E. Tyrrell J. Wood A. R. Beaumont R. N. Ruth K. S. Tuke M. A. (2016). Genome-wide association analyses in 128,266 individuals identifies new morningness and sleep duration loci. *PLoS Genet.* 12:e1006125. 10.1371/journal.pgen.1006125 27494321 PMC4975467

[B116] Juda M. Vetter C. Roenneberg T. (2013). Chronotype modulates sleep duration, sleep quality, and social jet lag in shift-workers. *J. Biol. Rhythms* 28 141–151. 10.1177/0748730412475042 23606613

[B117] Kalmbach D. A. Schneider L. D. Cheung J. Bertrsand S. J. Kariharan T. Pack A. I. (2017). Genetic basis of chronotype in humans: insights from three landmark GWAS. *Sleep* 40:2. 10.1093/sleep/zsw048 28364486 PMC6084759

[B118] Kandeger A. Egilmez U. Sayin A. A. Selvi Y. (2018). The relationship between night eating symptoms and disordered eating attitudes via insomnia and chronotype differences. *Psychiatry Res.* 268 354–357. 10.1016/j.psychres.2018.08.003 30098543

[B119] Kandeger A. Selvi Y. Tanyer D. K. (2019). The effects of individual circadian rhythm differences on insomnia, impulsivity, and food addiction. *Eat. Weight Disord.* 24 47–55. 10.1007/s40519-018-0518-x 29856005

[B120] Kang J. I. Park C. I. Sohn S. Y. Kim H. W. Namkoong K. Kim S. J. (2015). Circadian preference and trait impulsivity, sensation-seeking and response inhibition in healthy young adults. *Chronobiol. Int.* 32 235–241. 10.3109/07420528.2014.965313 25286137

[B121] Kaufmann C. N. Gershon A. Depp C. A. Miller S. Zeitzer J. M. Ketter T. A. (2018). Daytime midpoint as a digital biomarker for chronotype in bipolar disorder. *J. Affect Disord.* 241 586–591. 10.1016/j.jad.2018.08.032 30172210 PMC6436809

[B122] Kervran C. Fatseas M. Serre F. Taillard J. Beltran V. Leboucher J. (2015). Association between morningness/eveningness, addiction severity and psychiatric disorders among individuals with addictions. *Psychiatry Res.* 229 1024–1030. 10.1016/j.psychres.2015.05.026 26250146

[B123] Khan W. A. A. Conduit R. Kennedy G. A. Jackson M. L. (2020). The relationship between shift-work, sleep, and mental health among paramedics in Australia. *Sleep Health* 6 330–337. 10.1016/j.sleh.2019.12.002 32223969

[B124] Kilicaslan E. E. (2020). Associations between neurological soft signs, chronotype and sleep quality in schizophrenia. *Dusunen Adam* 2020:91. 10.14744/dajpns.2020.00091

[B125] Kim K. M. Han S. M. Heo K. Kim W. J. Chu M. K. (2020). Sex differences in the association between chronotype and risk of depression. *Sci. Rep.* 10:18512. 10.1038/s41598-020-75724-z 33116223 PMC7595163

[B126] Kitamura S. Hida A. Watanabe M. Enomoto M. Aritake-Okada S. Moriguchi Y. (2010). Evening preference is related to the incidence of depressive states independent of sleep-wake conditions. *Chronobiol. Int.* 27 1797–1812. 10.3109/07420528.2010.516705 20969524

[B127] Kivela L. Papadopoulos M. R. Antypa N. (2018). Chronotype and Psychiatric Disorders. *Curr. Sleep Med. Rep.* 4 94–103. 10.1007/s40675-018-0113-8 29888167 PMC5972175

[B128] Klein D. N. Kotov R. Bufferd S. J. (2011). Personality and depression: explanatory models and review of the evidence. *Annu. Rev. Clin. Psychol.* 7 269–295. 10.1146/annurev-clinpsy-032210-104540 21166535 PMC3518491

[B129] Koo D. L. Yang K. I. Kim J. H. Kim D. Sunwoo J. S. Hwangbo Y. (2021). Association between morningness-eveningness, sleep duration, weekend catch-up sleep and depression among Korean high-school students. *J. Sleep Res.* 30:e13063. 10.1111/jsr.13063 32391631

[B130] Kripke D. F. Klimecki W. T. Nievergelt C. M. Rex K. M. Murray S. S. Shekhtman T. (2014). Circadian polymorphisms in night owls, in bipolars, and in non-24-hour sleep cycles. *Psychiatry Investig.* 11 345–362. 10.4306/pi.2014.11.4.345 25395965 PMC4225198

[B131] Labonte B. Engmann O. Purushothaman I. Menard C. Wang J. Tan C. (2017). Sex-specific transcriptional signatures in human depression. *Nat. Med.* 23 1102–1111. 10.1038/nm.4386 28825715 PMC5734943

[B132] Lack L. Bailey M. Lovato N. Wright H. (2009). Chronotype differences in circadian rhythms of temperature, melatonin, and sleepiness as measured in a modified constant routine protocol. *Nat. Sci. Sleep* 1 1–8. 23616692 10.2147/nss.s6234 PMC3630920

[B133] Lane J. M. Vlasac I. Anderson S. G. Kyle S. D. Dixon W. G. Bechtold D. A. (2016). Genome-wide association analysis identifies novel loci for chronotype in 100,420 individuals from the UK Biobank. *Nat. Commun.* 7:10889. 10.1038/ncomms10889 26955885 PMC4786869

[B134] Latif I. Hughes A. T. L. Bendall R. C. A. (2019). Positive and negative affect mediate the influences of a maladaptive emotion regulation strategy on sleep quality. *Front. Psychiatry* 10:628. 10.3389/fpsyt.2019.00628 31543841 PMC6730659

[B135] Lee A. Myung S. K. Cho J. J. Jung Y. J. Yoon J. L. Kim M. Y. (2017). Night shift work and risk of depression: meta-analysis of observational studies. *J. Korean Med. Sci.* 32 1091–1096. 10.3346/jkms.2017.32.7.1091 28581264 PMC5461311

[B136] Lemoine P. Zawieja P. Ohayon M. M. (2013). Associations between morningness/eveningness and psychopathology: an epidemiological survey in three in-patient psychiatric clinics. *J. Psychiatr. Res.* 47 1095–1098. 10.1016/j.jpsychires.2013.04.001 23628386

[B137] Lewis K. J. S. Richards A. Karlsson R. Leonenko G. Jones S. E. Jones H. J. (2020). Comparison of genetic liability for sleep traits among individuals with bipolar disorder i or ii and control participants. *JAMA Psychiatry* 77 303–310. 10.1001/jamapsychiatry.2019.4079 31751445 PMC6902167

[B138] Li J. Z. Bunney B. G. Meng F. Hagenauer M. H. Walsh D. M. Vawter M. P. (2013). Circadian patterns of gene expression in the human brain and disruption in major depressive disorder. *Proc. Natl. Acad. Sci. U S A* 110 9950–9955. 10.1073/pnas.1305814110 23671070 PMC3683716

[B139] Li T. Xie Y. Tao S. Yang Y. Xu H. Zou L. (2020). Chronotype, sleep, and depressive symptoms among chinese college students: a cross-sectional study. *Front. Neurol.* 11:592825. 10.3389/fneur.2020.592825 33391156 PMC7773835

[B140] Liberman A. R. Kwon S. B. Vu H. T. Filipowicz A. Ay A. Ingram K. K. (2017). Circadian clock model supports molecular link between per3 and human anxiety. *Sci. Rep.* 7:9893. 10.1038/s41598-017-07957-4 28860482 PMC5579000

[B141] Lin C. Y. Imani V. Griffiths M. D. Brostrom A. Nygardh A. Demetrovics Z. (2021). Temporal associations between morningness/eveningness, problematic social media use, psychological distress and daytime sleepiness: mediated roles of sleep quality and insomnia among young adults. *J. Sleep Res.* 30:e13076. 10.1111/jsr.13076 32406567

[B142] Linke M. Jankowski K. S. (2021). Chronotype in individuals with schizophrenia: a meta-analysis. *Schizophr. Res.* 235 74–79. 10.1016/j.schres.2021.07.020 34332427

[B143] Lipnevich A. A. Crede M. Hahn E. Spinath F. M. Roberts R. D. Preckel F. (2017). How distinctive are morningness and eveningness from the Big Five factors of personality? A meta-analytic investigation. *J. Pers. Soc. Psychol.* 112 491–509. 10.1037/pspp0000099 27977220

[B144] Logan R. W. Edgar N. Gillman A. G. Hoffman D. Zhu X. McClung C. A. (2015). Chronic stress induces brain region-specific alterations of molecular rhythms that correlate with depression-like behavior in mice. *Biol. Psychiatry* 78 249–258. 10.1016/j.biopsych.2015.01.011 25771506 PMC4509914

[B145] Logan R. W. McClung C. A. (2019). Rhythms of life: circadian disruption and brain disorders across the lifespan. *Nat. Rev. Neurosci.* 20 49–65. 10.1038/s41583-018-0088-y 30459365 PMC6338075

[B146] Logan R. W. Williams W. P. III McClung C. A. (2014). Circadian rhythms and addiction: mechanistic insights and future directions. *Behav. Neurosci.* 128 387–412. 10.1037/a0036268 24731209 PMC4041815

[B147] Lopez-Soto P. J. Fabbian F. Cappadona R. Zucchi B. Manfredini F. Garcia-Arcos A. (2019). Chronotype, nursing activity, and gender: a systematic review. *J. Adv. Nurs.* 75 734–748. 10.1111/jan.13876 30307057

[B148] Lunsford-Avery J. R. Pelletier-Baldelli A. Korenic S. A. Schiffman J. Ellman L. M. Jackson L. (2021). Eveningness chronotype preference among individuals at clinical high risk for psychosis. *Schizophr. Res.* 236 3–8. 10.1016/j.schres.2021.07.034 34358763 PMC8464500

[B149] Lyall L. M. Wyse C. A. Graham N. Ferguson A. Lyall D. M. Cullen B. (2018). Association of disrupted circadian rhythmicity with mood disorders, subjective wellbeing, and cognitive function: a cross-sectional study of 91105 participants from the UK Biobank. *Lancet Psychiatry* 5 507–514. 10.1016/S2215-0366(18)30139-1 29776774

[B150] Lynch C. J. Gunning F. M. Liston C. (2020). Causes and consequences of diagnostic heterogeneity in depression: paths to discovering novel biological depression subtypes. *Biol. Psychiatry* 88 83–94. 10.1016/j.biopsych.2020.01.012 32171465

[B151] Ma H. Y. Liu Z. F. Xu Y. F. Hu X. D. Sun N. Li X. R. (2019). The association study of CLOCK gene polymorphisms with antidepressant effect in Chinese with major depressive disorder. *Per. Med.* 16 115–122. 10.2217/pme-2018-0123 30569826

[B152] Malhi G. S. Mann J. J. (2018). Depression. *Lancet* 392 2299–2312. 10.1016/s0140-6736(18)31948-2 30396512

[B153] Manni R. De Icco R. Cremascoli R. Ferrera G. Furia F. Zambrelli E. (2016). Circadian phase typing in idiopathic generalized epilepsy: dim light melatonin onset and patterns of melatonin secretion-semicurve findings in adult patients. *Epilepsy Behav.* 61 132–137. 10.1016/j.yebeh.2016.05.019 27344501

[B154] Maruani J. Geoffroy P. A. (2019). Bright light as a personalized precision treatment of mood disorders. *Front. Psychiatry* 10:85. 10.3389/fpsyt.2019.00085 30881318 PMC6405415

[B155] Maukonen M. Havulinna A. S. Mannisto S. Kanerva N. Salomaa V. Partonen T. (2020). Genetic associations of chronotype in the finnish general population. *J. Biol. Rhythms.* 35 501–511. 10.1177/0748730420935328 32579418 PMC7534025

[B156] Mazri F. H. Manaf Z. A. Shahar S. Mat Ludin A. F. (2019). The association between chronotype and dietary pattern among adults: a scoping review. *Int. J. Environ. Res. Public Health* 17:1. 10.3390/ijerph17010068 31861810 PMC6981497

[B157] McCarthy M. J. Wei H. Nievergelt C. M. Stautland A. Maihofer A. X. Welsh D. K. (2019). Chronotype and cellular circadian rhythms predict the clinical response to lithium maintenance treatment in patients with bipolar disorder. *Neuropsychopharmacology* 44 620–628. 10.1038/s41386-018-0273-8 30487653 PMC6333516

[B158] McGlashan E. M. Drummond S. P. A. Cain S. W. (2018). Evening types demonstrate reduced SSRI treatment efficacy. *Chronobiol. Int.* 35 1175–1178. 10.1080/07420528.2018.1458316 29658803

[B159] McHill A. W. Sano A. Hilditch C. J. Barger L. K. Czeisler C. A. Picard R. (2021). Robust stability of melatonin circadian phase, sleep metrics, and chronotype across months in young adults living in real-world settings. *J. Pineal. Res.* 70:e12720. 10.1111/jpi.12720 33523499 PMC9135480

[B160] Melo M. C. Garcia R. F. Araujo C. F. Luz J. H. Bruin P. F. Bruin V. M. (2020). Chronotype in bipolar disorder: an 18-month prospective study. *Braz. J. Psychiatry* 42 68–71. 10.1590/1516-4446-2019-0489 31269097 PMC6986486

[B161] Melo M. C. A. Abreu R. L. C. Linhares Neto V. B. de Bruin P. F. C. de Bruin V. M. S. (2017). Chronotype and circadian rhythm in bipolar disorder: a systematic review. *Sleep Med. Rev.* 34 46–58. 10.1016/j.smrv.2016.06.007 27524206

[B162] Menke A. (2019). is the hpa axis as target for depression outdated, or is there a new hope?. *Front. Psychiatry* 10:101. 10.3389/fpsyt.2019.00101 30890970 PMC6413696

[B163] Merikanto I. Kronholm E. Peltonen M. Laatikainen T. Lahti T. Partonen T. (2012). Relation of chronotype to sleep complaints in the general finnish population. *Chronobiol. Int.* 29 311–317. 10.3109/07420528.2012.655870 22390244

[B164] Merikanto I. Kronholm E. Peltonen M. Laatikainen T. Vartiainen E. Partonen T. (2015). Circadian preference links to depression in general adult population. *J. Affect. Disord.* 188 143–148. 10.1016/j.jad.2015.08.061 26363264

[B165] Merikanto I. Partonen T. (2020). Increase in eveningness and insufficient sleep among adults in population-based cross-sections from 2007 to 2017. *Sleep Med.* 75 368–379. 10.1016/j.sleep.2020.07.046 32950882

[B166] Minelli A. Di Palma M. Rocchi M. B. L. Ponzio E. Barbadoro P. Bracci M. (2021). Cortisol, chronotype, and coping styles as determinants of tolerance of nursing staff to rotating shift work. *Chronobiol. Int.* 38 666–680. 10.1080/07420528.2021.1887883 33827343

[B167] Minors D. S. Waterhouse J. M. Wirz-Justice A. (1991). A human phase-response curve to light. *Neurosci. Lett.* 133 36–40. 10.1016/0304-3940(91)90051-t 1791996

[B168] Mokros L. Nowakowska-Domagala K. Koprowicz J. Witusik A. Pietras T. (2021). The association between chronotype and suicidality among students of the medicine and psychology faculties - the mediating role of general mental health indices. *Chronobiol. Int.* 38 509–517. 10.1080/07420528.2020.1865393 33397172

[B169] Morris D. W. Trivedi M. H. Fava M. Wisniewski S. R. Balasubramani G. K. Khan A. Y. (2009). Diurnal mood variation in outpatients with major depressive disorder. *Depress Anxiety* 26 851–863. 10.1002/da.20557 19306304

[B170] Mrazek D. A. Hornberger J. C. Altar C. A. Degtiar I. (2014). A review of the clinical, economic, and societal burden of treatment-resistant depression: 1996-2013. *Psychiatr. Serv.* 65 977–987. 10.1176/appi.ps.201300059 24789696

[B171] Najem J. Saber M. Aoun C. El Osta N. Papazian T. Rabbaa Khabbaz L. (2020). Prevalence of food addiction and association with stress, sleep quality and chronotype: a cross-sectional survey among university students. *Clin. Nutr.* 39 533–539. 10.1016/j.clnu.2019.02.038 30878156

[B172] Ng T. H. Chung K. F. Ng T. K. Lee C. T. Chan M. S. (2016). Correlates and prognostic relevance of sleep irregularity in inter-episode bipolar disorder. *Compr. Psychiatry* 69 155–162. 10.1016/j.comppsych.2016.05.016 27423356

[B173] Norbury R. (2021). Diurnal preference and depressive symptomatology: a meta-analysis. *Sci. Rep.* 11:12003. 10.1038/s41598-021-91205-3 34099766 PMC8184740

[B174] Oliveira T. Marinho V. Carvalho V. Magalhaes F. Rocha K. Ayres C. (2018). Genetic polymorphisms associated with circadian rhythm dysregulation provide new perspectives on bipolar disorder. *Bipolar. Disord.* 20 515–522. 10.1111/bdi.12624 29441659

[B175] Onat O. E. Kars M. E. Gul S. Bilguvar K. Wu Y. Ozhan A. (2020). Human CRY1 variants associate with attention deficit/hyperactivity disorder. *J. Clin. Invest.* 130 3885–3900. 10.1172/JCI135500 32538895 PMC7324179

[B176] Ozdogan M. G. Aydin E. F. Ustundag M. F. Ceyhun H. A. Oral E. Bakan E. (2020). Homocysteine, chronotype and clinical course in bipolar disorder patients. *Nord. J. Psychiatry* 74 340–345. 10.1080/08039488.2019.1710250 31900022

[B177] Park Y. M. (2020). Relationship between Auditory Evoked Potentials and Circadian Preference in Patients with Major Depressive Episodes. *Brain Sci.* 10:6. 10.3390/brainsci10060370 32545632 PMC7349307

[B178] Passos G. S. Santana M. G. Poyares D. D’Aurea C. V. Teixeira A. A. Tufik S. (2017). Chronotype and anxiety are associated in patients with chronic primary insomnia. *Braz. J. Psychiatry* 39 183–186. 10.1590/1516-4446-2016-2007 28076650 PMC7111444

[B179] Preckel F. Lipnevich A. A. Schneider S. Roberts R. D. (2011). Chronotype, cognitive abilities, and academic achievement: a meta-analytic investigation. *Learn. Indiv. Diff.* 21 483–492. 10.1016/j.lindif.2011.07.003

[B180] Przepiorka A. Blachnio A. Cudo A. (2021). Relationships between morningness, Big Five personality traits, and problematic internet use in young adult university students: mediating role of depression. *Chronobiol. Int.* 38 248–259. 10.1080/07420528.2020.1851703 33317359

[B181] Randler C. Bilger S. (2009). Associations among sleep, chronotype, parental monitoring, and pubertal development among German adolescents. *J. Psychol.* 143 509–520. 10.3200/JRL.143.5.509-520 19943401

[B182] Randler C. Horzum M. B. Vollmer C. (2013). Internet addiction and its relationship to chronotype and personality in a turkish university student sample. *Soc. Sci. Comp. Rev.* 32 484–495. 10.1177/0894439313511055

[B183] Randler C. Schaal S. (2010). Morningness-eveningness, habitual sleep-wake variables and cortisol level. *Biol. Psychol.* 85 14–18. 10.1016/j.biopsycho.2010.04.006 20450953

[B184] Razavi P. Devore E. E. Bajaj A. Lockley S. W. Figueiro M. G. Ricchiuti V. (2019). Shift work, chronotype, and melatonin rhythm in nurses. *Cancer Epidemiol. Biomarkers Prev.* 28 1177–1186. 10.1158/1055-9965.EPI-18-1018 31142495 PMC6750706

[B185] Rodriguez-Cortes F. J. Morales-Cane I. Rodriguez-Munoz P. M. Cappadona R. Giorgi A. De Manfredini R. (2022). Individual circadian preference, eating disorders and obesity in children and adolescents: a dangerous liaison? a systematic review and a meta-analysis. *Children* 9:2. 10.3390/children9020167 35204888 PMC8870066

[B186] Roenneberg T. Kuehnle T. Pramstaller P. P. Ricken J. Havel M. Guth A. (2004). A marker for the end of adolescence. *Curr. Biol.* 14 R1038–R1039. 10.1016/j.cub.2004.11.039 15620633

[B187] Roenneberg T. Merrow M. (2016). The circadian clock and human health. *Curr. Biol.* 26 R432–R443. 10.1016/j.cub.2016.04.011 27218855

[B188] Roenneberg T. Pilz L. K. Zerbini G. Winnebeck E. C. (2019). Chronotype and social jetlag: a (self-) critical review. *Biology* 8:3. 10.3390/biology8030054 31336976 PMC6784249

[B189] Roenneberg T. Wirz-Justice A. Merrow M. (2003). Life between clocks: daily temporal patterns of human chronotypes. *J. Biol. Rhythms* 18 80–90. 10.1177/0748730402239679 12568247

[B190] Romo-Nava F. Blom T. J. Cuellar-Barboza A. B. Winham S. J. Colby C. L. Nunez N. A. (2020a). Evening chronotype as a discrete clinical subphenotype in bipolar disorder. *J. Affect. Disord.* 266 556–562. 10.1016/j.jad.2020.01.151 32056926

[B191] Romo-Nava F. Blom T. J. Guerdjikova A. Winham S. J. Cuellar-Barboza A. B. Nunez N. A. (2020b). Evening chronotype, disordered eating behavior, and poor dietary habits in bipolar disorder. *Acta Psychiatr. Scand.* 142 58–65. 10.1111/acps.13179 32335894

[B192] Rosenberg J. Maximov M. II Reske F. G. Shah N. J. (2014). Early to bed, early to rise”: diffusion tensor imaging identifies chronotype-specificity. *Neuroimage* 84 428–434. 10.1016/j.neuroimage.2013.07.086 24001455

[B193] Rumble M. E. Dickson D. McCall W. V. Krystal A. D. Case D. Rosenquist P. B. (2018). The relationship of person-specific eveningness chronotype, greater seasonality, and less rhythmicity to suicidal behavior: a literature review. *J. Affect. Disord.* 227 721–730. 10.1016/j.jad.2017.11.078 29179142 PMC5805608

[B194] Sack R. L. Brandes R. W. Kendall A. R. Lewy A. J. (2000). Entrainment of free-running circadian rhythms by melatonin in blind people. *N. Engl. J. Med.* 343 1070–1077. 10.1056/NEJM200010123431503 11027741

[B195] Sakurai T. (2007). The neural circuit of orexin (hypocretin): maintaining sleep and wakefulness. *Nat. Rev. Neurosci.* 8 171–181. 10.1038/nrn2092 17299454

[B196] Salfi F. D’Atri A. Tempesta D. Ferrara M. (2021). Sleeping under the waves: a longitudinal study across the contagion peaks of the COVID-19 pandemic in Italy. *J. Sleep Res.* 2021:e13313. 10.1111/jsr.13313 33687798 PMC8250209

[B197] Salk R. H. Hyde J. S. Abramson L. Y. (2017). Gender differences in depression in representative national samples: meta-analyses of diagnoses and symptoms. *Psychol. Bull.* 143 783–822. 10.1037/bul0000102 28447828 PMC5532074

[B198] Scammell T. E. Arrigoni E. Lipton J. O. (2017). Neural Circuitry of Wakefulness and Sleep. *Neuron* 93 747–765. 10.1016/j.neuron.2017.01.014 28231463 PMC5325713

[B199] Schmidt C. Collette F. Reichert C. F. Maire M. Vandewalle G. Peigneux P. (2015). Pushing the limits: chronotype and time of day modulate working memory-dependent cerebral activity. *Front. Neurol.* 6:199. 10.3389/fneur.2015.00199 26441819 PMC4585243

[B200] Shareh H. Eshaghi Sani M. (2019). Predictive role of morningness-eveningness personality, cognitive flexibility and cognitive emotion regulation in marital satisfaction in middle-aged women. *Iran. J. Psychiatry Clin. Psychol.* 2019 384–399. 10.32598/ijpcp.24.4.384

[B201] Shen G. H. Alloy L. B. Abramson L. Y. Sylvia L. G. (2008). Social rhythm regularity and the onset of affective episodes in bipolar spectrum individuals. *Bipolar. Disord.* 10 520–529. 10.1111/j.1399-5618.2008.00583.x 18452448 PMC4090015

[B202] Shi S. Q. White M. J. Borsetti H. M. Pendergast J. S. Hida A. Ciarleglio C. M. (2016). Molecular analyses of circadian gene variants reveal sex-dependent links between depression and clocks. *Transl. Psychiatry* 6:e748. 10.1038/tp.2016.9 26926884 PMC4872462

[B203] Siemiginowska P. Iskra-Golec I. (2020). Blue light effect on EEG activity - the role of exposure timing and chronotype. *Light. Res. Technol.* 52 472–484. 10.1177/1477153519876969

[B204] Silva A. Dos Santos M. J. Goes Gitai D. L. de Miranda Coelho J. A. P. de Andrade T. G. (2020). Depression and anxiety symptoms correlate with diurnal preference, sleep habits, and Per3 VNTR polymorphism (rs57875989) in a non-clinical sample. *J. Affect. Disord.* 277 260–270. 10.1016/j.jad.2020.07.138 32841827

[B205] Silva V. M. Magalhaes J. E. M. Duarte L. L. (2020). Quality of sleep and anxiety are related to circadian preference in university students. *PLoS One* 15:e0238514. 10.1371/journal.pone.0238514 32877438 PMC7467298

[B206] Simor P. Zavecz Z. Palosi V. Torok C. Koteles F. (2015). The influence of sleep complaints on the association between chronotype and negative emotionality in young adults. *Chronobiol. Int.* 32 1–10. 10.3109/07420528.2014.935786 25003651

[B207] Simpkin C. T. Jenni O. G. Carskadon M. A. Wright K. P. Jr. Akacem L. D. Garlo K. G. (2014). Chronotype is associated with the timing of the circadian clock and sleep in toddlers. *J. Sleep. Res.* 23 397–405. 10.1111/jsr.12142 24628737 PMC4117798

[B208] Smith C. S. Reilly C. Midkiff K. (1989). Evaluation of three circadian rhythm questionnaires with suggestions for an improved measure of morningness. *J. Appl. Psychol.* 74 728–738. 10.1037/0021-9010.74.5.728 2793773

[B209] Song J. Feng P. Zhao X. Xu W. Xiao L. Zhou J. (2018). Chronotype regulates the neural basis of response inhibition during the daytime. *Chronobiol. Int.* 35 208–218. 10.1080/07420528.2017.1392550 29144173

[B210] Spulber S. Conti M. DuPont C. Raciti M. Bose R. Onishchenko N. (2015). Alterations in circadian entrainment precede the onset of depression-like behavior that does not respond to fluoxetine. *Transl Psychiatry* 5 e603. 10.1038/tp.2015.94 26171984 PMC5068723

[B211] Stewart R. Besset A. Bebbington P. Brugha T. Lindesay J. Jenkins R. (2006). Insomnia comorbidity and impact and hypnotic use by age group in a national survey population aged 16 to 74 years. *Sleep* 29 1391–1397. 10.1093/sleep/29.11.1391 17162985

[B212] Stolarski M. Jankowski K. S. Matthews G. Kawalerczyk J. (2016). Wise “birds” follow their clock: the role of emotional intelligence and morningness-eveningness in diurnal regulation of mood. *Chronobiol. Int.* 33 51–63. 10.3109/07420528.2015.1115413 26730807

[B213] Sun H. Tan Q. Fan G. Tsui Q. (2014). Different effects of rumination on depression: key role of hope. *Int. J. Ment. Health Syst.* 8:53. 10.1186/1752-4458-8-53 25926875 PMC4414423

[B214] Sun J. Chen M. Cai W. Wang Z. Wu S. Sun X. (2019). Chronotype: implications for sleep quality in medical students. *Chronobiol. Int.* 36 1115–1123. 10.1080/07420528.2019.1619181 31140322

[B215] Sun X. Gustat J. Bertisch S. M. Redline S. Bazzano L. (2020). The association between sleep chronotype and obesity among black and white participants of the Bogalusa Heart Study. *Chronobiol. Int.* 37 123–134. 10.1080/07420528.2019.1689398 31747792 PMC6981036

[B216] Suzuki M. Dallaspezia S. Locatelli C. Lorenzi C. Uchiyama M. Colombo C. (2017). CLOCK gene variants associated with the discrepancy between subjective and objective severity in bipolar depression. *J. Affect. Disord.* 210 14–18. 10.1016/j.jad.2016.12.007 27992853

[B217] Taillard J. Philip P. Bioulac B. (1999). Morningness/eveningness and the need for sleep. *J. Sleep Res.* 8 291–295. 10.1046/j.1365-2869.1999.00176.x 10646169

[B218] Taillard J. Philip P. Chastang J. F. Bioulac B. (2004). Validation of Horne and Ostberg morningness-eveningness questionnaire in a middle-aged population of French workers. *J. Biol. Rhythms* 19 76–86. 10.1177/0748730403259849 14964706

[B219] Taillard J. Philip P. Chastang J. F. Diefenbach K. Bioulac B. (2001). Is self-reported morbidity related to the circadian clock? *J. Biol. Rhythms* 16 183–190. 10.1177/074873001129001764 11302560

[B220] Taillard J. Philip P. Claustrat B. Capelli A. Coste O. Chaumet G. (2011). Time course of neurobehavioral alertness during extended wakefulness in morning- and evening-type healthy sleepers. *Chronobiol. Int.* 28 520–527. 10.3109/07420528.2011.590623 21797780

[B221] Taillard J. Philip P. Coste O. Sagaspe P. Bioulac B. (2003). The circadian and homeostatic modulation of sleep pressure during wakefulness differs between morning and evening chronotypes. *J. Sleep. Res.* 2003:369. 10.1046/j.0962-1105.2003.00369.x 14633238

[B222] Taillard J. Sagaspe P. Philip P. Bioulac S. (2021). Sleep timing, chronotype and social jetlag: impact on cognitive abilities and psychiatric disorders. *Biochem. Pharmacol.* 191:114438. 10.1016/j.bcp.2021.114438 33545116

[B223] Takaesu Y. (2018). Circadian rhythm in bipolar disorder: a review of the literature. *Psychiatry Clin. Neurosci.* 72 673–682. 10.1111/pcn.12688 29869403

[B224] Takeuchi H. Taki Y. Sekiguchi A. Nouchi R. Kotozaki Y. Nakagawa S. (2015). Regional gray matter density is associated with morningness–eveningness: evidence from voxel-based morphometry. *Neuroimage* 117 294–304. 10.1016/j.neuroimage.2015.05.037 26003859

[B225] Tan M. N. Mevsim V. Pozlu Cifci M. Sayan H. Ercan A. E. Ergin O. F. (2020). Who is happier among preclinical medical students: the impact of chronotype preference. *Chronobiol. Int.* 37 1163–1172. 10.1080/07420528.2020.1761373 32396746

[B226] Taylor B. J. Bowman M. A. Brindle A. Hasler B. P. Roecklein K. A. Krafty R. T. (2020). Evening chronotype, alcohol use disorder severity, and emotion regulation in college students. *Chronobiol. Int.* 37 1725–1735. 10.1080/07420528.2020.1800028 32791860 PMC10080672

[B227] Taylor B. J. Hasler B. P. (2018). Chronotype and mental health: recent advances. *Curr. Psychiatry Rep.* 20:59. 10.1007/s11920-018-0925-8 30039327

[B228] Teixeira G. P. Balieiro L. C. T. Gontijo C. A. Fahmy W. M. Maia Y. C. P. Crispim C. A. (2020). The association between chronotype, food craving and weight gain in pregnant women. *J. Hum. Nutr. Diet.* 33 342–350. 10.1111/jhn.12723 31800138

[B229] Thapa N. Kim B. Yang J. G. Park H. J. Jang M. Son H. E. (2020). The relationship between chronotype, physical activity and the estimated risk of dementia in community-dwelling older adults. *Int. J. Environ. Res. Public Health* 2020:17. 10.3390/ijerph17103701 32456356 PMC7277473

[B230] Tian Y. Chen X. Xu D. Yu J. Lei X. (2020). Connectivity within the default mode network mediates the association between chronotype and sleep quality. *J. Sleep Res.* 29:e12948. 10.1111/jsr.12948 31793113

[B231] Toda M. Kawai T. Takeo K. Rokutan K. Morimoto K. (2013). Associations between chronotype and salivary endocrinological stress markers. *Endocr. Res.* 38 1–7. 10.3109/07435800.2012.683225 22591393

[B232] Tonetti L. Fabbri M. Martoni M. Natale V. (2011). Season of birth and sleep-timing preferences in adolescents. *Chronobiol. Int.* 28 536–540. 10.3109/07420528.2011.590261 21797782

[B233] Tonon A. C. Carissimi A. Schimitt R. L. de Lima L. S. Pereira F. D. S. Hidalgo M. P. (2020). How do stress, sleep quality, and chronotype associate with clinically significant depressive symptoms? a study of young male military recruits in compulsory service. *Braz. J. Psychiatry* 42 54–62. 10.1590/1516-4446-2018-0286 31166545 PMC6986495

[B234] Torquati L. Mielke G. I. Brown W. J. Burton N. W. Kolbe-Alexander T. L. (2019). Shift work and poor mental health: a meta-analysis of longitudinal studies. *Am. J. Public Health* 109 e13–e20. 10.2105/AJPH.2019.305278 31536404 PMC6775929

[B235] Tosuntas S. B. Balta S. Emirtekin E. Kircaburun K. Griffiths M. D. (2018). Adolescents’ eveningness chronotype and cyberbullying perpetration: the mediating role of depression-related aggression and anxiety-related aggression. *Biolog. Rhyt. Res.* 51 40–50. 10.1080/09291016.2018.1513132

[B236] Touitou Y. Touitou D. Reinberg A. (2016). Disruption of adolescents’ circadian clock: the vicious circle of media use, exposure to light at night, sleep loss and risk behaviors. *J. Physiol. Paris* 110(4 Pt B), 467–479. 10.1016/j.jphysparis.2017.05.001 28487255

[B237] Turkoglu G. Selvi Y. (2020). The relationship between chronotype, sleep disturbance, severity of fibromyalgia, and quality of life in patients with fibromyalgia. *Chronobiol. Int.* 37 68–81. 10.1080/07420528.2019.1684314 31687843

[B238] Uzer A. Yucens B. (2020a). Chronotype and depressive symptoms in healthy subjects: the mediating role of hopelessness and subjective sleep quality. *Chronobiol. Int.* 37 1173–1180. 10.1080/07420528.2020.1775629 32635745

[B239] Uzer A. Yucens B. (2020b). The effect of circadian preferences on insomnia severity and depressive symptoms via sleep hygiene in older adults with depression and healthy controls. *Psychogeriatrics* 20 871–879. 10.1111/psyg.12610 32954590

[B240] Van den Berg J. F. Kivela L. Antypa N. (2018). Chronotype and depressive symptoms in students: an investigation of possible mechanisms. *Chronobiol. Int.* 35 1248–1261. 10.1080/07420528.2018.1470531 29764217

[B241] Vollmer C. Michel U. Randler C. (2012). Outdoor light at night (LAN) is correlated with eveningness in adolescents. *Chronobiol. Int.* 29 502–508. 10.3109/07420528.2011.635232 22214237

[B242] Walker W. H. Borniger J. C. Gaudier-Diaz M. M. Hecmarie Melendez-Fernandez O. Pascoe J. L. Courtney DeVries A. (2020). Acute exposure to low-level light at night is sufficient to induce neurological changes and depressive-like behavior. *Mol. Psychiatry* 25 1080–1093. 10.1038/s41380-019-0430-4 31138889 PMC6881534

[B243] Walsh N. A. Repa L. M. Garland S. N. (2021). Mindful larks and lonely owls: The relationship between chronotype, mental health, sleep quality, and social support in young adults. *J. Sleep Res.* 2021:e13442. 10.1111/jsr.13442 34272788

[B244] Wang H. Lane J. M. Jones S. E. Dashti H. S. Ollila H. M. Wood A. R. (2019). Genome-wide association analysis of self-reported daytime sleepiness identifies 42 loci that suggest biological subtypes. *Nat. Commun.* 10:3503. 10.1038/s41467-019-11456-7 31409809 PMC6692391

[B245] Watts A. L. Norbury R. (2017). Reduced effective emotion regulation in night owls. *J. Biol. Rhythms* 32 369–375. 10.1177/0748730417709111 28627300

[B246] Weiss C. Woods K. Filipowicz A. Ingram K. K. (2020). Sleep quality, sleep structure, and per3 genotype mediate chronotype effects on depressive symptoms in young adults. *Front. Psychol.* 11:2028. 10.3389/fpsyg.2020.02028 32982844 PMC7479229

[B247] Wills C. Ghani S. Tubbs A. Fernandez F. X. Athey A. Turner R. (2021). Chronotype and social support among student athletes: impact on depressive symptoms. *Chronobiol. Int.* 2021 1–11. 10.1080/07420528.2021.1927072 34039131

[B248] Wirz-Justice A. Benedetti F. (2020). Perspectives in affective disorders: Clocks and sleep. *Eur. J. Neurosci.* 51 346–365. 10.1111/ejn.14362 30702783

[B249] Wirz-Justice A. Skene D. J. Munch M. (2021). The relevance of daylight for humans. *Biochem. Pharmacol.* 191:114304. 10.1016/j.bcp.2020.114304 33129807

[B250] Wittmann M. Dinich J. Merrow M. Roenneberg T. (2006). Social jetlag: misalignment of biological and social time. *Chronobiol. Int.* 23 497–509. 10.1080/07420520500545979 16687322

[B251] Wong M. L. Kling M. A. Munson P. J. Listwak S. Licinio J. Prolo P. (2000). Pronounced and sustained central hypernoradrenergic function in major depression with melancholic features: relation to hypercortisolism and corticotropin-releasing hormone. *Proc. Natl. Acad. Sci. U S A* 97 325–330. 10.1073/pnas.97.1.325 10618417 PMC26662

[B252] Xiao H. Cai H. Li X. (2021). Non-visual effects of indoor light environment on humans: a review(). *Physiol. Behav.* 228:113195. 10.1016/j.physbeh.2020.113195 33022281

[B253] Xu N. Shinohara K. Saunders K. E. A. Geddes J. R. Cipriani A. (2021). Effect of lithium on circadian rhythm in bipolar disorder: a systematic review and meta-analysis. *Bipolar. Disord.* 2021:13070. 10.1111/bdi.13070 33650218

[B254] Yang Y.-T. Kaplan K. A. Zeitzer J. M. (2020). A comparison of sleep, depressive symptoms, and parental perceptions between U.S. and Taiwan adolescents with self-reported sleep problems. *Sleep Adv.* 2020: zaa004. 10.1093/sleepadvances/zpaa004/5905468 PMC7731573 33345187

[B255] Yeom J. W. Jeong S. Seo J. Y. Jeon S. Lee H. J. (2020). Association of the serotonin 2a receptor rs6311 polymorphism with diurnal preference in koreans. *Psychiatry Investig.* 17 1137–1142. 10.30773/pi.2020.0358 33115187 PMC7711123

[B256] You M. Laborde S. Dosseville F. Salinas A. Allen M. S. (2020). Associations of chronotype, Big Five, and emotional competences with perceived stress in university students. *Chronobiol. Int.* 37 1090–1098. 10.1080/07420528.2020.1752705 32400200

[B257] Zaki N. F. Sabri Y. M. Farouk O. Abdelfatah A. Spence D. W. Bahammam A. S. (2020). Depressive symptoms, sleep profiles and serum melatonin levels in a sample of breast cancer patients. *Nat. Sci. Sleep* 12 135–149. 10.2147/NSS.S206768 32104121 PMC7025675

[B258] Zavada A. Gordijn M. C. Beersma D. G. Daan S. Roenneberg T. (2005). Comparison of the munich chronotype questionnaire with the horne-ostberg’s morningness-eveningness score. *Chronobiol. Int.* 22 267–278. 10.1081/cbi-200053536 16021843

[B259] Zerbini G. Kantermann T. Merrow M. (2020). Strategies to decrease social jetlag: reducing evening blue light advances sleep and melatonin. *Eur. J. Neurosci.* 51 2355–2366. 10.1111/ejn.14293 30506899

[B260] Zerbini G. Winnebeck E. C. Merrow M. (2021). Weekly, seasonal, and chronotype-dependent variation of dim-light melatonin onset. *J. Pineal. Res.* 70:e12723. 10.1111/jpi.12723 33608951

[B261] Zhou J. Hsiao F. C. Shi X. Yang J. Huang Y. Jiang Y. (2021). Chronotype and depressive symptoms: a moderated mediation model of sleep quality and resilience in the 1st-year college students. *J. Clin. Psychol.* 77 340–355. 10.1002/jclp.23037 32761628

[B262] Zhou L. Zhang H. Luo Z. Liu X. Yang L. Hu H. (2020). Abnormal night sleep duration and inappropriate sleep initiation time are associated with elevated anxiety symptoms in chinese rural adults: the henan rural cohort. *Psychiatry Res.* 291:113232. 10.1016/j.psychres.2020.113232 32574900

[B263] Zimmermann L. K. (2011). Chronotype and the transition to college life. *Chronobiol. Int.* 28 904–910. 10.3109/07420528.2011.618959 22080735

[B264] Zuraikat F. M. St-Onge M. P. Makarem N. Boege H. L. Xi H. Aggarwal B. (2021). Evening chronotype is associated with poorer habitual diet in us women, with dietary energy density mediating a relation of chronotype with cardiovascular health. *J. Nutr.* 151 1150–1158. 10.1093/jn/nxaa442 33758908 PMC8112764

