# Supplementary material for: Chronotype, circadian rhythm, and psychiatric disorders: Recent evidence and potential mechanisms
Source: Front Neurosci. 2022 Aug 10;16:811771. doi: 10.3389/fnins.2022.811771 (PMC9399511; doi:10.3389/fnins.2022.811771)
Supplement: Supplementary file 1 [file Presentation_1.pdf]

The MEQ, developed in the 1970s by Horne and Östberg, is the most commonly used instrument for assessing chronotype and show satisfactory reliability and high stability [1]. The MEQ consists of 19 items assessing personal preference for physical, intellectual, and social activities as well as subjectively performing better or worse during the daytime. Most studies using MEQ scores divide chronotype into five types [1]. Some studies use percentiles for classification [2]. The rMEQ was developed by Adan and Almirall because of the unsuitability of the length of the MEQ in some situations. The MCTQ, developed by Roenneberg, is based on the assessment of waking and sleeping times on weekdays and weekends (free days) and is the most recent instrument measuring chronotype [3]. After exploring sleep onset and wake-up latency, the midpoint of sleep on free days (MSF) whose definition is the midpoint between sleep onset and offset on free days, is calculated. The most frequent MSF occurs at 4:14 a.m. [4]. There are consistent classification outcomes based on the MEQ or the MCTQ. Besides, the classification of chronotype for MSF showed a strong association with MEQ scores [5]. However, the two instruments are assessing different aspects of chronotype (the MEQ is influenced by psychological and social factors, whereas the MCTQ reflects the internal biological clock, the sleep-wake cycle) [6; 7]. The MEQ tends to evaluate an individual's preference for performing various daily activities, but the MCTQ emphasizes the preference for sleep-related behavior [8]. The selection of the two instruments depends on the research questions. Here, we reviewed the three most common questionnaires currently used for research on chronotypes, including the evaluation dimensions and the classification criteria (Table 1).

Reference

[1] J. Horne, and O.J.I.j.o.c. Ostberg. A self-assessment questionnaire to determine morningness-eveningness in human circadian rhythms[J]. 1976, 4(2): 97-110

[2] S. Kitamura, A. Hida, M. Watanabe, M. Enomoto, S. Aritake-Okada, Y. Moriguchi, Y. Kamei, and K. Mishima. Evening preference is related to the incidence of depressive states independent of sleep-wake conditions[J]. Chronobiol Int, 2010, 27(9-10): 1797-1812.http://dx.doi.org/10.3109/07420528.2010.516705

[3] T. Roenneberg, A. Wirz-Justice, and M. Merrow. Life between Clocks: Daily Temporal Patterns of Human Chronotypes[J]. Journal of Biological Rhythms, 2003, 18(1): 80-90.http://dx.doi.org/10.1177/0748730402239679

[4] T. Roenneberg, T. Kuehnle, M. Juda, T. Kantermann, K. Allebrandt, M. Gordijn, and M. Merrow. Epidemiology of the human circadian clock[J]. Sleep Med Rev, 2007, 11(6): 429-438.http://dx.doi.org/10.1016/j.smrv.2007.07.005

[5] A. Zavada, M.C. Gordijn, D.G. Beersma, S. Daan, and T. Roenneberg. Comparison of the Munich Chronotype Questionnaire with the Horne-Ostberg's Morningness-Eveningness Score[J]. Chronobiol Int, 2005, 22(2): 267-278.http://dx.doi.org/10.1081/cbi-200053536

[6] M.J. Muller, and A. Haag. The concept of chronotypes and its clinical importance for depressive disorders[J]. Int J Psychiatry Med, 2018, 53(3): 224-240.http://dx.doi.org/10.1177/0091217417749787

[7] T. Roenneberg. What is chronotype?[J]. Sleep and Biological Rhythms, 2012, 10(2): 75-76.http://dx.doi.org/10.1111/j.1479-8425.2012.00541.x

[8] S. Bauducco, C. Richardson, and M. Gradisar. Chronotype, circadian rhythms and mood[J]. Curr Opin Psychol, 2020, 34: 77-83.http://dx.doi.org/10.1016/j.copsyc.2019.09.002
