# Supplementary material for: Chronotype, circadian rhythm, and psychiatric disorders: Recent evidence and potential mechanisms
Source: Front Neurosci. 2022 Aug 10;16:811771. doi: 10.3389/fnins.2022.811771 (PMC9399511; doi:10.3389/fnins.2022.811771)
Supplement: Supplementary file 2 [file Table_1.pdf]

Table Questionnaires for chronotype

Name	Questions	Variables	Evaluation & Classification
MEQ	19 items	• Sleep and wake-up times<br>• Preferred times for physical and Mental activity<br>• Subjective alertness	Definitely morning type 70-86<br>Moderately morning type 59-69<br>Intermediate type 42-58<br>Moderately evening type 31-41<br>Definitely evening type 16-30
MCTQ	29 items	**(workdays and work-free days)**<br>• So_w/SO_f<br>• GU_w/GU_f<br>• SD_w/SD_f<br>• TBT_w/TBT_f<br>• MSW/MSF	**MSFsc**<br>Extreme early type<br>Moderate early type<br>Slight early type<br>Normal type<br>Slight late type<br>Moderate late type<br>Extreme late type
CSM	13 items	• Activity planning<br>• Morning alertness<br>• Evening alertness	Morning Type 44-55<br>Intermediate Type 23-43<br>Evening Type 13-22

SO: sleep onset; GU: local time of getting out of bed; SD: sleep duration; TBT: total time in bed; MS: mid-sleep. MSFsc: the mid-sleep on work-free days corrected for "oversleep" due to the sleep debt accumulated during the workdays, calculation: If $SD_f \leq SD_w$, MSFsc = MSF or If $SD_f > SD_w$, MSFsc = MSF - $(SD_f - SD_w)/2$. The categorization of chronotype by using MCTQ is based on quantile of the MSFsc scores in samples.
